# Artesunate Inhibits the Growth Behavior of Docetaxel-Resistant Prostate Cancer Cells

**DOI:** 10.3389/fonc.2022.789284

**Published:** 2022-02-07

**Authors:** Olesya Vakhrusheva, Holger H. H. Erb, Vitus Bräunig, Sascha D. Markowitsch, Patricia Schupp, Patrick C. Baer, Kimberly Sue Slade, Anita Thomas, Igor Tsaur, Martin Puhr, Zoran Culig, Jindrich Cinatl, Martin Michaelis, Thomas Efferth, Axel Haferkamp, Eva Juengel

**Affiliations:** ^1^ Department of Urology and Pediatric Urology, University Medical Center Mainz, Mainz, Germany; ^2^ Department of Urology, University of Dresden, Dresden, Germany; ^3^ Department of Internal Medicine III, Nephrology, University Hospital, Goethe-University, Frankfurt am Main, Germany; ^4^ Department of Urology, Medical University of Innsbruck, Innsbruck, Austria; ^5^ Institute of Medical Virology, Goethe-University, Frankfurt am Main, Germany; ^6^ Industrial Biotechnology Centre and School of Biosciences, University of Kent, Canterbury, United Kingdom; ^7^ Institute of Pharmaceutical and Biomedical Sciences, Johannes Gutenberg University Mainz, Mainz, Germany

**Keywords:** prostate cancer (PCa), docetaxel (DX) resistance, artesunate (ART), Traditional Chinese Medicine (TCM), growth inhibition, apoptosis, ferroptosis

## Abstract

Novel therapeutic strategies are urgently needed for advanced metastatic prostate cancer (PCa). Phytochemicals used in Traditional Chinese Medicine seem to exhibit tumor suppressive properties. Therefore, the therapeutic potential of artesunate (ART) on the progressive growth of therapy-sensitive (parental) and docetaxel (DX)-resistant PCa cells was investigated. Parental and DX-resistant PCa cell lines DU145, PC3, and LNCaP were incubated with artesunate (ART) [1-100 µM]. ART-untreated and ‘non-cancerous’ cells served as controls. Cell growth, proliferation, cell cycle progression, cell death and the expression of involved proteins were evaluated. ART, dose- and time-dependently, significantly restricted cell growth and proliferation of parental and DX-resistant PCa cells, but not of ‘normal, non-cancerous’ cells. ART-induced growth and proliferation inhibition was accompanied by G0/G1 phase arrest and down-regulation of cell cycle activating proteins in all DX-resistant PCa cells and parental LNCaP. In the parental and DX-resistant PC3 and LNCaP cell lines, ART also promoted apoptotic cell death. Ferroptosis was exclusively induced by ART in parental and DX-resistant DU145 cells by increasing reactive oxygen species (ROS). The anti-cancer activity displayed by ART took effect in all three PCa cell lines, but through different mechanisms of action. Thus, in advanced PCa, ART may hold promise as a complementary treatment together with conventional therapy.

## 1 Introduction

Prostate carcinoma (PCa) is the most common cancer in men without curative therapy for metastatic disease, although diverse therapeutic strategies have contributed to prolonged survival and reduced therapy-induced complications (1–3). The chemotherapeutic agent docetaxel (DX) is one of the most effective drugs used to treat metastatic PCa (4). However, the efficacy of the DX-based therapy is limited to only a few months, due to therapy resistance (5–7). The sequential treatment with second-line therapeutics remains less effective (8). Due to existing and emerging resistance to DX, innovative therapeutic approaches are essential in bringing new effective treatment options to patients with castration- and chemotherapy-resistant PCa.

In the past decades, the demand for complementary and alternative medicine (CAM) has increased and is reflected by its use among cancer patients, ranging from 30 - 90%, depending on the country, culture, cancer type and stage (9–11). Like pharmacological agents, combination therapy with natural compounds requires thorough investigation to increase efficacy while minimizing side effects. Since valid scientific investigation regarding the anti-tumor activity of natural compounds is frequently lacking, contraindications to their use cannot be ruled out (12, 13).

**Figure 1:**
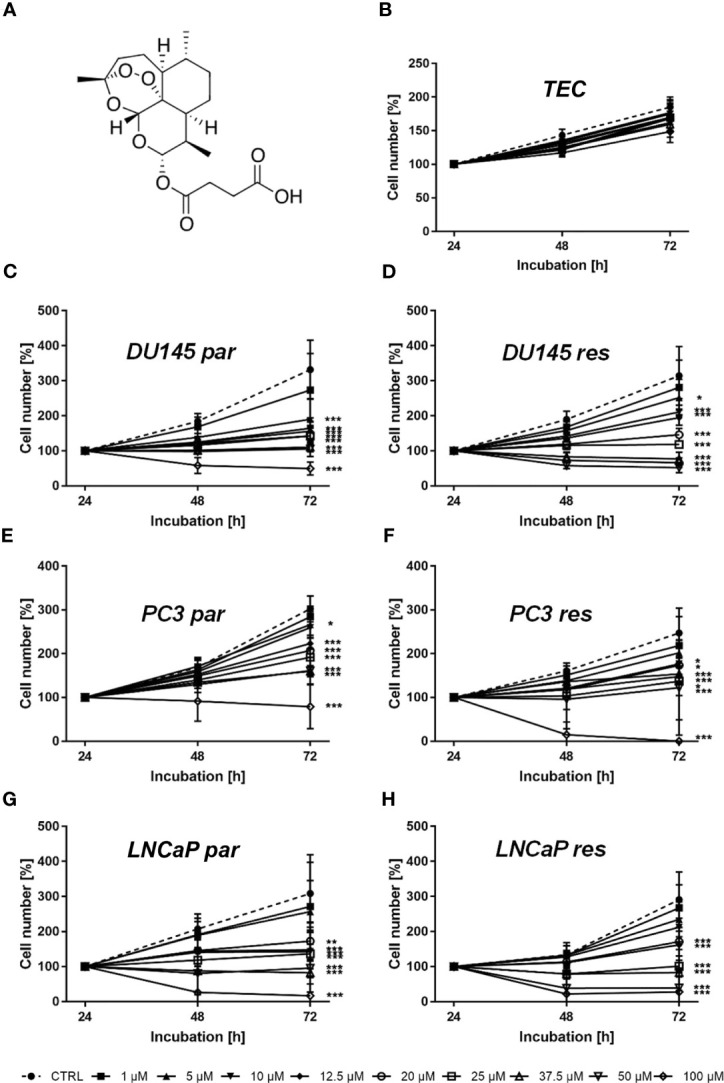
Chemical structure of ART **(A)** and cell growth of normal (healthy) TEC cells **(B)** as well asof parental (par) and DX-resistant (res) DU145 **(C, D)**, PC3 **(E, F)**, and LNCaP **(G, H)** PCa cells after 24, 48, and 72 h incubation with ART concentrations ascending from 1 – 100 µM. Untreated cells served as controls. Cell number was set to 100% after 24 h incubation. Error bars indicate standard deviation (SD). Significant difference to untreated control: *p ≤ .05, **p ≤ .01, ***p ≤ .001. n = 3.

Artemisinin, extracted from Artemisia annua (Sweet Wormwood), was developed in Traditional Chinese Medicine to treat malaria (14). Artesunate (ART) (**Figure 1A**), derived from artemisinin, exhibits remarkable anti-cancer activity towards a broad variety of tumor cell lines (15, 16), including prostate carcinoma (17–19). Moreover, ART exhibits anti-inflammatory properties, thereby preventing tissue destruction induced by carcinogens *in vivo* (20). An endoperoxide moiety of ART reacts with iron, leading to the formation of cytotoxic radicals (21). Cancer cells have elevated iron and transferrin receptor levels, compared to normal cells, thus, making them susceptible to reactive oxygen species (ROS) (22–24). In doxorubicin-resistant T-cell leukemia and cisplatin-resistant neuroblastoma, ART has been shown to trigger ROS formation, resulting in apoptosis induction (25, 26). ART has also caused cell cycle arrest in ovarian cancer cells *via* increased ROS generation (27). Besides initiating apoptotic cell death, ART induces ferroptosis, an iron-dependent programmed cell death in head and neck cancer (28). The resulting ART-mediated decrease in cellular glutathione (GSH) and accumulation of lipid ROS levels abrogated cisplatin resistance in these cells. In addition, ART activates ferroptosis in pancreatic carcinoma cells bearing oncogenic Ras (29). The anti-cancer activity of ART is therefore multifaceted. The goal of the present study was to investigate ART’s mode of action in a panel of therapy-sensitive (parental) and DX-resistant prostate cancer cells.

## 2 Materials and Methods

### 2.1 Isolation and Cultivation of Primary Human Renal Tubular Epithelial Cells

Primary human renal tubular epithelial cells (TEC) served as ‘normal, non-cancerous’ control cells. TEC were separated, cultured and characterized as described previously (30, 31). In brief, cells were isolated after tumor nephrectomies from renal tissue not involved in renal cell carcinoma. The donors gave written informed consent. Ethical standards were complied with as defined by the World Medical Association Declaration of Helsinki. The tissue was disintegrated using crossed blades, digested with collagenase/dispase, and passed through a 106 µm mesh. Remaining cohered cells were then incubated with collagenase IV, DNase and MgCl_2_ and further purified by Percoll density gradient centrifugation (31). After centrifugation, the fraction between 1.05 and 1.076 g/ml was collected and washed twice in three volumes of ice cold HBSS (Gibco, Thermo Fisher Scientific, Darmstadt, Germany). Isolated cells were seeded in 6-well plates. Medium 199 (M4530, Sigma, Taufkirchen, Germany) with a physiologic glucose concentration (100 mg/dl) was supplemented with 10% fetal bovine serum (FBS; Biochrom, Berlin, Germany), used as standard culture medium and replaced every three to four days. Confluent cells were passaged by trypsinization. Cells between passages 2 and 5 were used for the experiments.

### 2.2 Cell Lines

The PCa cell lines DU145, PC3, and LNCaP were purchased from the German Collection of Microorganisms and Cell Cultures (DSMZ). The DX-resistant sublines were derived from the Resistant Cancer Cell Line (RCCL) collection (http://research.kent.ac.uk/industrial-biotechnology-centre/the-resistant-cancer-cell-line-rccl-collection/). Drug-adapted cancer sublines were established by continuous exposure to stepwise increasing drug concentrations as previously described (32, 33). DX-sensitive (parental) DU145, PC3, and their respective DX-resistant sublines were cultivated in RPMI-1640 medium (Gibco, Thermo Fisher Scientific, Darmstadt, Germany). Parental LNCaP and the DX-resistant subline were sub-cultured in Iscove Basal medium (Biochrom GmbH, Berlin, Germany). Media were supplemented with 10% fetal calf serum (FCS) (Gibco, Thermo Fisher Scientific, Darmstadt, Germany), 1% glutamax (Gibco^®^, Thermo Fisher Scientific, Darmstadt, Germany), and 1% Anti/Anti (Gibco, Thermo Fisher Scientific, Darmstadt, Germany). 20 mM HEPES-buffer (Sigma-Aldrich, Darmstadt, Germany) was added to the RPMI-1640 medium. The DX-resistant sublines were exposed to 12.5 nM DX (Sigma-Aldrich, Darmstadt, Germany) three times a week. All cell lines were cultivated in a humidified, 5% CO_2_ incubator.

### 2.3 Drug Treatment

ART (Sigma-Aldrich, Darmstadt, Germany), dissolved in DMSO, was applied for 24, 48, or 72 h at a concentration of 1-100 μM. Controls (parental and cisplatin-resistant) remained ART-untreated. To evaluate toxic effects of ART, cell viability was determined by trypan blue (Sigma-Aldrich, Darmstadt, Germany). Ferrostatin-1 (Sigma-Aldrich, Darmstadt, Germany), the ferroptosis inhibitor, was used at a concentration of 20 μM.

### 2.4 Cell Growth and Proliferation

Cell growth was defined using 3-(4,5-dimethylthiazol- 2-yl)-2,5-diphenyltetrazolium bromide (MTT) dye. PCa DX-sensitive and DX-resistant cells (50 µL, 1 × 10^5^ cells/mL) were seeded into 96-well-plates. ART (Sigma-Aldrich, Darmstadt, Germany) was applied for 24, 48, and 72 h at a concentration of 1-100 µM. Then, MTT (0.5 mg/mL) (Sigma-Aldrich, Darmstadt, Germany) was added. After 4 h incubation with MTT, cells were lysed with 100 µL solubilization buffer per well containing 10% SDS in 0.01 M HCl. The plates were then incubated overnight at 37°C in a 5% CO_2_ incubator. Absorbance at 570 nm was determined for each well using a multi-mode microplate-reader (Tecan, Spark 10 M, Crailsheim, Germany). After subtracting background absorbance and offsetting with a standard curve, results were expressed as mean cell number in percent. To illustrate dose-response kinetics, the mean cell number after 24 h incubation was set to 100%. Each experiment was done in triplicate.

Cell proliferation was measured using a BrdU (Bromodeoxyuridine/5-bromo-2′-deoxyuridine) cell proliferation enzyme-linked immunosorbent assay (ELISA) kit (Calbiochem/Merck Biosciences, Darmstadt, Germany). Cells (50 µL, 1 × 10^5^ cells/mL) were seeded into 96-well-plates and incubated with ART for 48 h at concentrations from 12.5 to 100 µM. 20 µL BrdU-labeling solution per well was added 24 h prior to fixation and staining using anti-BrdU mAb, according to the manufacturer’s protocol. Absorbance was measured at 450 nm using a multi-mode microplate-reader (Tecan, Spark 10 M, Crailsheim, Germany). Values presented as percentage compared to untreated controls were set to 100%.

### 2.5 Cell Cycle Analysis

To investigate cell cycle progression of ART-treated and control PCa cells, 1 × 10^6^ cells were stained with propidium iodide (PI) (50 µg/mL) (Invitrogen, Thermo Fisher Scientific, Darmstadt, Germany) and analyzed by flow cytometry (Fortessa X20, BD Biosciences, Heidelberg, Germany). Data acquisition was carried out using DIVA software (BD Biosciences, Heidelberg, Germany), and cell cycle phase distribution was analyzed by ModFit LT 5.0 software (Verity Software House, Topsham, ME, USA). The number of cells in the G0/G1, S, or G2/M phases was expressed as a percentage.

### 2.6 Cell Death (Apoptosis, Necrosis, Ferroptosis)

The FITC-Annexin V Apoptosis Detection kit (BD Biosciences, Heidelberg, Germany) was used to quantify apoptotic and necrotic events. After washing cells twice with PBS, 1 × 10^5^ cells were resuspended in 500 µL of 1 × binding buffer and incubated with 5 µL Annexin V-FITC and/or 5 µL PI in the dark for 15 min. Staining was measured by flow cytometer (Fortessa X20, BD Biosciences, Heidelberg, Germany). The percentage of apoptotic and necrotic cells was calculated using DIVA software (BD Biosciences, Heidelberg, Germany). Further analysis was done by FlowJo software (BD Biosciences, Heidelberg, Germany).

To evaluate ferroptosis, cells were treated with 37.5 µM ART or ART combined with the ferroptosis inhibitor ferrostatin-1 [20 µM] (Sigma-Aldrich, Darmstadt, Germany) for 24 and 48 h. Ferroptosis was assessed using BrdU cell proliferation enzyme-linked immunosorbent assay (ELISA) kit (Calbiochem/Merck Biosciences, Darmstadt, Germany), as described above. For more details, see *Cell Growth and Proliferation* (2.4).

### 2.7 Western Blot Analysis of Cell Cycle and Cell Death Regulating Proteins

The expression and activity of cell cycle and cell death regulating proteins were explored by Western blot analysis. Tumor cell lysates (50 µg) were applied to 10 or 12% polyacrylamide gel and separated for 10 min at 80 V and 1 h at 120 V. The protein was then transferred to nitrocellulose membranes (1 h, 100 V). After blocking with 10% non-fat dry milk for 1 h, the membranes were incubated overnight with the following primary antibodies directed against cell cycle regulating proteins: CDK1 (Mouse IgG1, clone 2), CDK2 (Mouse IgG2a, clone 55), cyclin A (Mouse IgG1, clone 25), cyclin B (Mouse IgG1, clone 18), and cyclin D1 (Rabbit IgG, clone 92G2), (all: BD Biosciences, Heidelberg, Germany).

To indicate apoptosis and ferroptosis-related proteins, the following primary antibodies against the corresponding proteins (total expression) were used: caspase 3 (Rabbit, pAb), caspase 8 (Rabbit IgG, clone D35G2), PARP-1 (Rabbit IgG, clone 46D11), (all Cell Signaling, Frankfurt am Main, Germany), and GPX4 (Rabbit IgG, ab41787, Abcam, Berlin, Germany). HRP-conjugated rabbit-anti-mouse IgG or goat-anti-rabbit IgG served as secondary antibodies (IgG, both: dilution 1:1000, Dako, Glostrup, Denmark). The membranes were incubated 2 min with an ECL detection reagent (AC2204, Azure Biosystems, Munich, Germany) to visualize proteins with a Sapphire Imager (Azure Biosystems, Munich, Germany). Protein expression was normalized to total protein. To quantify total protein all membranes were stained with Coomassie brilliant blue and measured by Sapphire Imager. AlphaView software (ProteinSimple, San Jose, CA, USA) was used for pixel density analysis of the protein bands. The ratio of protein intensity/β-actin intensity or whole protein intensity was calculated and expressed in percentage, related to the untreated control, set to 100%.

### 2.8 GSH Assay

The GSH level was evaluated using the GSH-Glo™ Glutathione Assay (Promega GmbH, Walldorf, Germany). 5 × 10^3^ cells/well were seeded into a 96-well plate and incubated for 24 h with 37.5 µM ART. Experiments were performed according to the manufacturer’s protocol. Luminescence was measured using a multi-mode microplate-reader (Tecan, Spark 10 M, Tecan, Grödig, Austria).

### 2.9 Statistical Analysis

All experiments were performed at least three times. The evaluation and generation of mean values as well as normalization in percent were done with Microsoft Excel. The standard deviation and statistical significance was calculated with GraphPad Prism 7.0 (GraphPad Software Inc., San Diego, CA, USA): two-sided *t*-Test (Western blot, apoptosis, cell cycle), one-way ANOVA test (BrdU), and two-way ANOVA test (MTT). Correction for multiple comparisons was done using the conservative Bonferroni method. Differences were considered statistically significant at a *p*-value ≤.05.

## 3 Results

### 3.1 ART Inhibits Cell Growth and Proliferation of Parental and DX-Resistant PCa Cells

Tumor cell growth and proliferation of parental and DX-resistant PCa cells as well as of ‘normal/non-tumor’ cells were evaluated after ART application. Growth of ART-treated ‘normal’ cells, here primary human renal tubular epithelial cells (TEC), remained unchanged, compared to the untreated cells (**Figure 1B**). In contrast, ART induced a significant time- and dose-dependent growth inhibition in parental and DX-resistant DU145, PC3, and LNCaP cells (**Figures 1C–H**). Parental and DX-resistant DU145 and LNCaP cells showed similar growth inhibitory effects after 72 h exposure to ART (**Figures 1C, D, G, H** **)**. In contrast, DX-resistant PC3 cells responded more sensitively to ART than their parental counterparts (**Figures 1E, F** **)**. Both parental and DX-resistant DU145 cells revealed first significant growth inhibition after 72 h at an ART concentration of 5 µM (**Figures 1C, D** **)**. Parental PC3 cells also showed growth inhibition at 5 µM ART. DX-resistant PC3 cells first displayed growth inhibition at 10 µM ART, but showed a total response, leaving no more cells at the highest concentration of 100 µM (**Figure 1F**). In parental PC3 cells, 100 µM ART induced a decrease in cell number but did not lead to the death of all cells (**Figure 1E**), as seen with the DX-resistant PC3 cells (**Figure 1F**). LNCaP cells, both parental and DX-resistant, responded with a significant reduction of tumor cell growth at 20 µM ART. Since treatment with 37.5 µM showed a strong growth inhibitory effect in all tested cell lines, 37.5 µM ART and higher concentrations were used in the following experiments.

**Figure 2:**
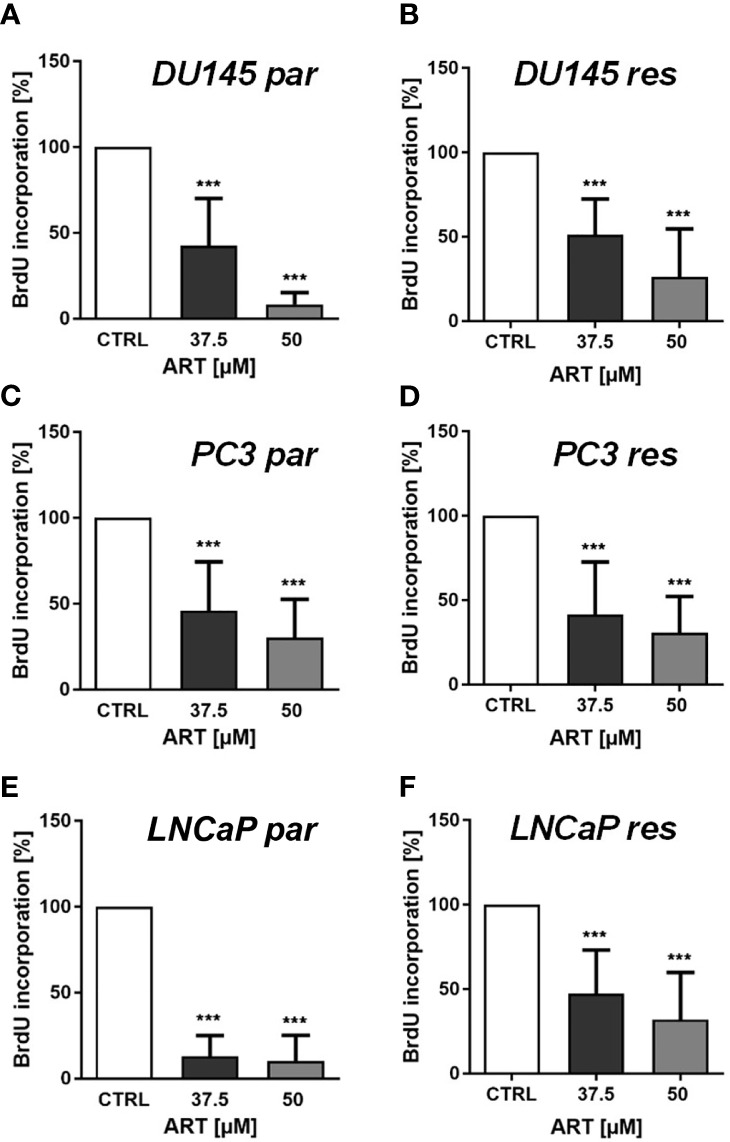
Tumor cell proliferation of parental (par) and DX-resistant (res) DU145 **(A, B)**, PC3 **(C, D)**, and LNCaP **(E, F)** cells incubated for 48 h with ART [37.5 and 50 µM]. Untreated controls were set to 100%. Error bars indicate standard deviation (SD). Significant difference to untreated control: ***p ≤ .001. n = 3.

In line with the growth data, proliferation of all parental and DX-resistant PCa cells was significantly diminished in a dose-dependent manner after exposure to ART for 48 h (**Figures 2A–F**).

### 3.2 ART Induces Cell Cycle Arrest, Accompanied by Alterations in the Expression of Cell Cycle Regulating Proteins

**Figure 3:**
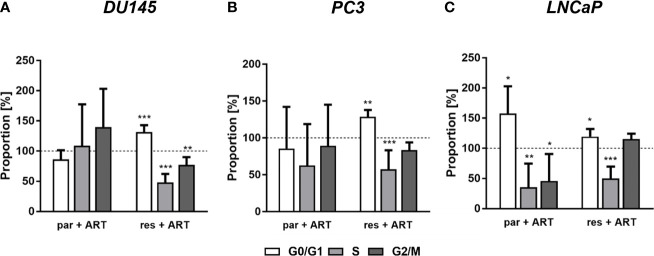
Distribution of cell cycle phases: proportion of parental (par) and DX-resistant (res) PCa cells, DU145 **(A)**, PC3 **(B)**, and LNCaP **(C)**, in the G0/G1, S, and G2/M phases after 48 h ART application [37.5 µM]. Untreated cells served as controls (dotted line set to 100%). Error bars indicate standard deviation (SD). Significant difference to untreated control: *p ≤.05, **p ≤.01, ***p ≤.001. n = 3.

In parental and DX-resistant PCa cells, ART-mediated cell growth inhibition and reduced proliferation were associated with impaired cell cycle progression. ART caused a significant G0/G1 phase arrest in DX-resistant DU145, PC3, and LNCaP cells (**Figures 3A–C**). The increase in G0/G1 phase was connected with a significant reduction in S phase (all) and G2/M phase (DU145) cells (**Figures 3A–C**). Furthermore, ART resulted in a significant gain of G0/G1 phase cells and a simultaneous decrease of S and G2/M phase cells in parental LNCaP cells (**Figure 1C**). In contrast, parental DU145 and PC3 cells showed no significant changes in cell cycle progression after treatment with ART (**Figures 3A, B** **)**.

**Figure 4:**
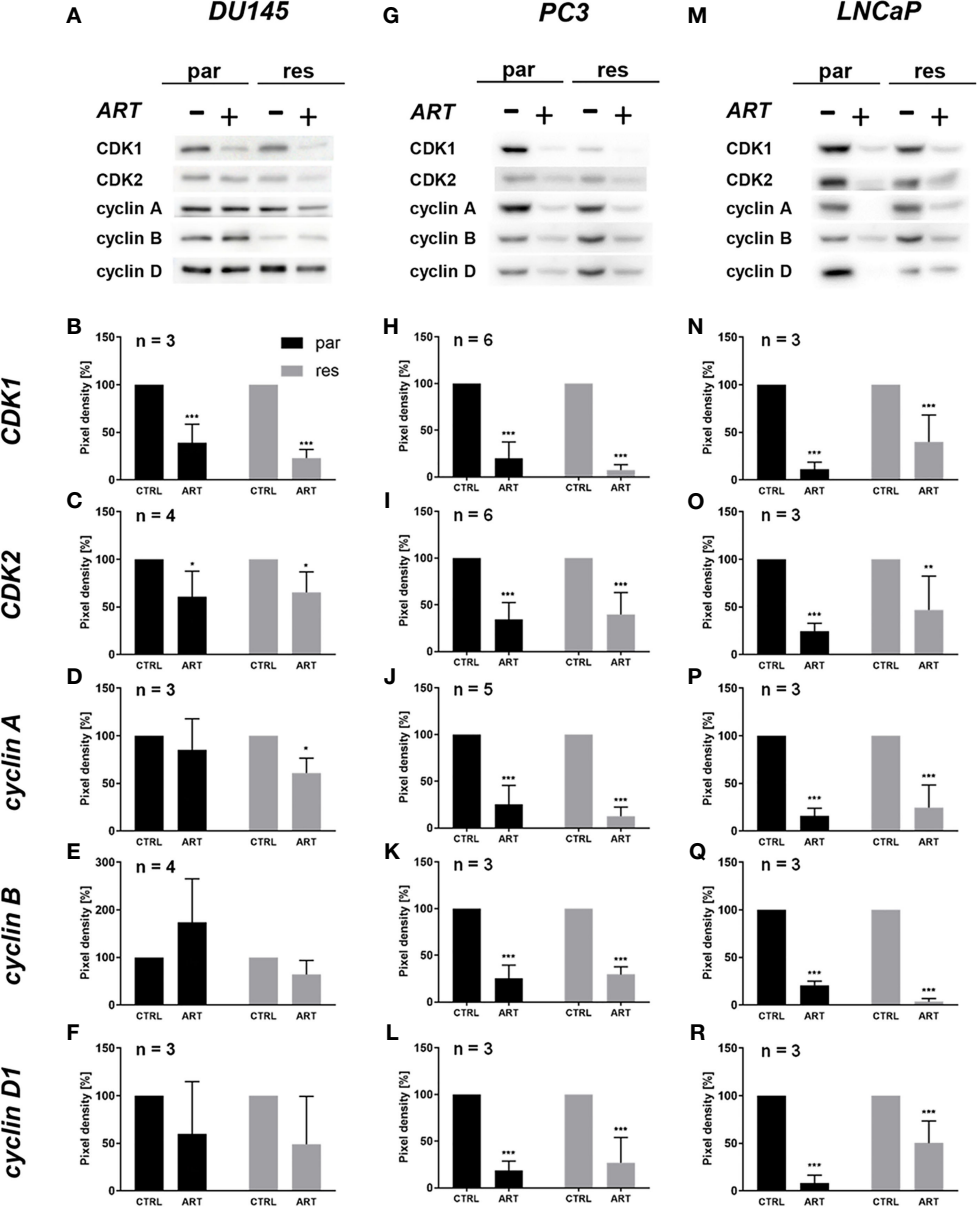
Expression of cell cycle regulating proteins: representative Western blot images of cell cycle regulating proteins in parental (par) and DX-resistant (res) DU145 **(A)**, PC3 **(G)**, and LNCaP **(M)** cells with (+) and without (-) ART. Pixel density analysis of the expression of cell cycle regu-lating proteins CDK1 **(B, H, N)**, CDK2 **(C, I, O)**, cyclin A **(D, J, P)**, cyclin B **(E, K, Q)** and cyclin D1 **(F, L, R)** in parental (par) and DX-resistant (res) cells after 48 h exposure to ART [37.5 µM], compared to untreated controls (set to 100%). Analysis of pixel density was normalized by a total protein staining. Error bars indicate standard deviation (SD). Significant difference to untreated control: *p ≤.05, **p ≤.01, ***p ≤.001. n = 3. For detailed information regarding the Western blots, see **Figures S1.1–15**.

The G0/G1 cell cycle arrest in the PCa cells was accompanied by significantly diminished expression of regulating CDK-cyclin complexes (**Figure 4**). In DX-resistant DU145, DX-resistant PC3, and both parental and DX-resistant LNCaP cells, the cell cycle proteins responsible for successful S and G2/M phase progression, CDK1 (**Figures 4A, B, G, H, M, N** and **Figure S1.1, 1.6, 1.11**) and CDK2 (**Figures 4A, C, G, I, M, O** and **Figure S1.2, 1.7, 1.12**), as well as cyclin A (**Figures 4A, D, G, J, M, P** and **Figure S1.3, 1.8, 1.13**) were significantly down-regulated in response to ART. Moreover, DX-resistant PC3 and parental and DX-resistant LNCaP cells revealed a significant decrease of cyclin B (**Figures 4G, K, M, Q** and **Figure S1.9, 1.14**), involved in G2/M phase progression, and cyclin D1 (**Figures 4G, L, M, R** and **Figure S1.10, 1.15**), responsible for G0/G1 phase progression. Also in the parental DU145 and PC3 cells expression of some activating proteins were impaired, however without affecting cell cycle regulation.

### 3.3 Artesunate Induces Apoptotic Cell Death in PCa Cells

**Figure 5:**
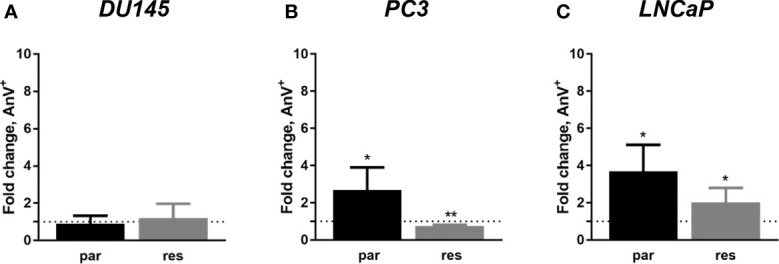
Apoptosis induction: parental (par) and DX-resistant (res) DU145 **(A)**, PC3 **(B)**, and LNCaP **(C)** cells treated for 48 h with ART [37.5 µM]. The analysis was performed by Annexin V (AnV+)/PI detection (in fold change). Untreated cells served as controls (dotted line, set to 1). Error bars indicate standard deviation (SD). Significant difference to untreated control: *p ≤.05, **p ≤.01. n = 3.

**Figure 6:**
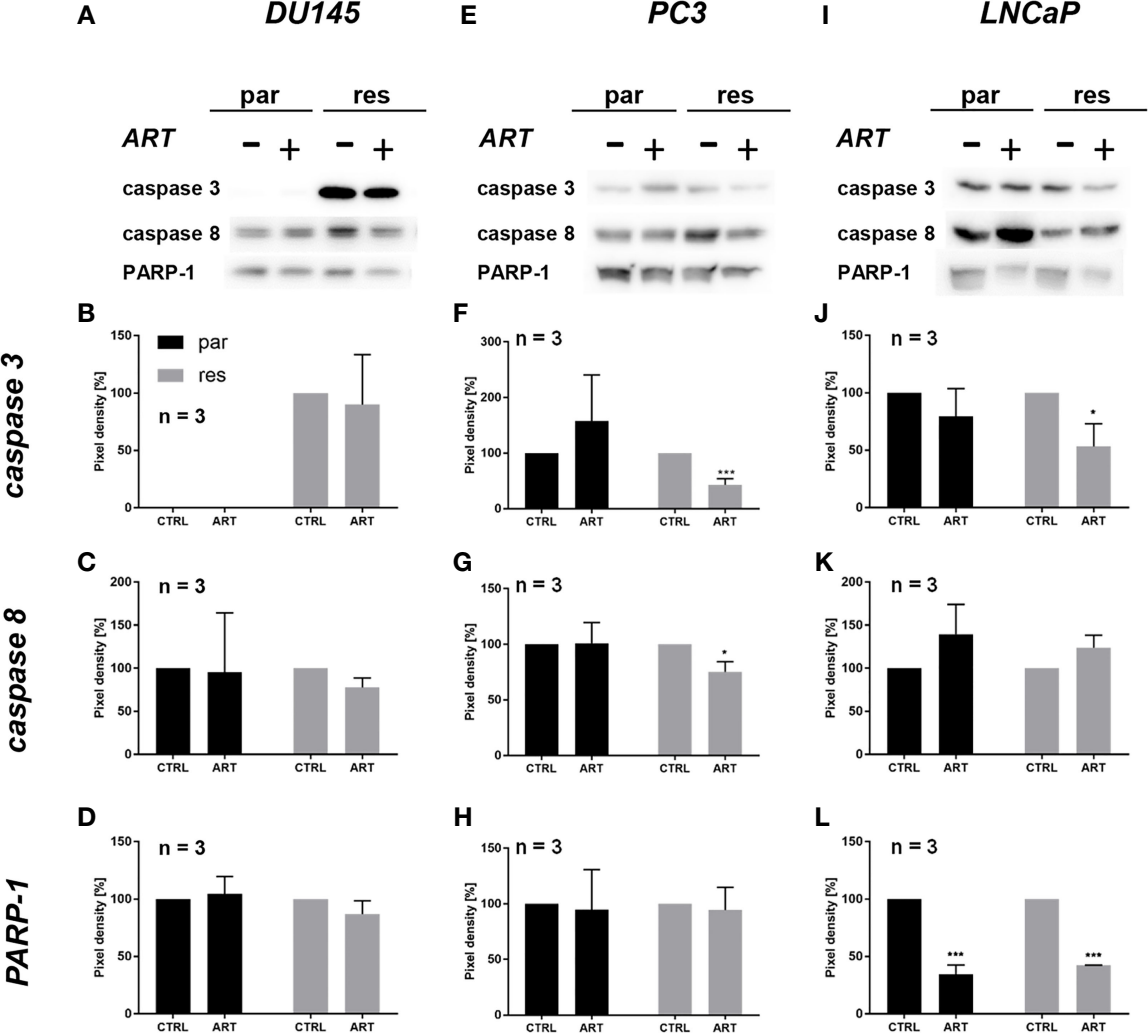
Apoptotic signaling: representative Western blot images of apoptosis-related proteins in parental (par) and DX-resistant (res) DU145 **(A)**, PC3 **(E)** and LNCaP **(I)** cells with (+) and without (-) ART. Pixel density of caspase 3 **(B, F, J)**, caspase 8 **(C, G, K)** and PARP-1 **(D, H, L)** expression in parental (par) and DX-resistant (res) cells. All protein analysis was normalized by a total protein control. Untreated cells served as controls (100%). Error bars indicate standard deviation (SD). Significant difference indicated by: *p ≤ .05, ***p ≤ .001. n = 3. For detailed information regarding the Western blots, see **Figures S2.1–9**.

Since changes in cell cycle progression after ART treatment could not explain the observed growth and proliferation inhibition in all three PCa cell lines, apoptosis was assessed. No apoptotic events were detected after exposure to ART in parental and DX-resistant DU145 cells, compared to the untreated control (**Figure 5A**). This was verified by no significant alteration in the expression of PARP-1 and effector caspases 3 and 8 (**Figures 6A–D** and **Figure S2.1–3**). A significant elevation of apoptotic cells was detected in parental PC3 cells (**Figure 5B**) even though no significant changes were detected in the chosen proteins responsible for apoptotic signaling (**Figures 6E–H** and **Figure S2.4–6**). An increase in apoptotic events was observed in both parental and DX-resistant LNCaP cells (**Figure 5C**). Here, activated apoptotic signaling was confirmed by a significant down-regulation of caspase 3 in DX-resistant LNCaP cells and PARP-1 in both parental and DX-resistant LNCaP cells after exposure to ART (**Figures 6I–L** and **Figure S2.7–9**). Necrotic events were generally low and remained unchanged after ART treatment (data not shown).

### 3.4 Artesunate Induces Ferroptosis in DU145 But Not in PC3 or LNCaP Cells

**Figure 7:**
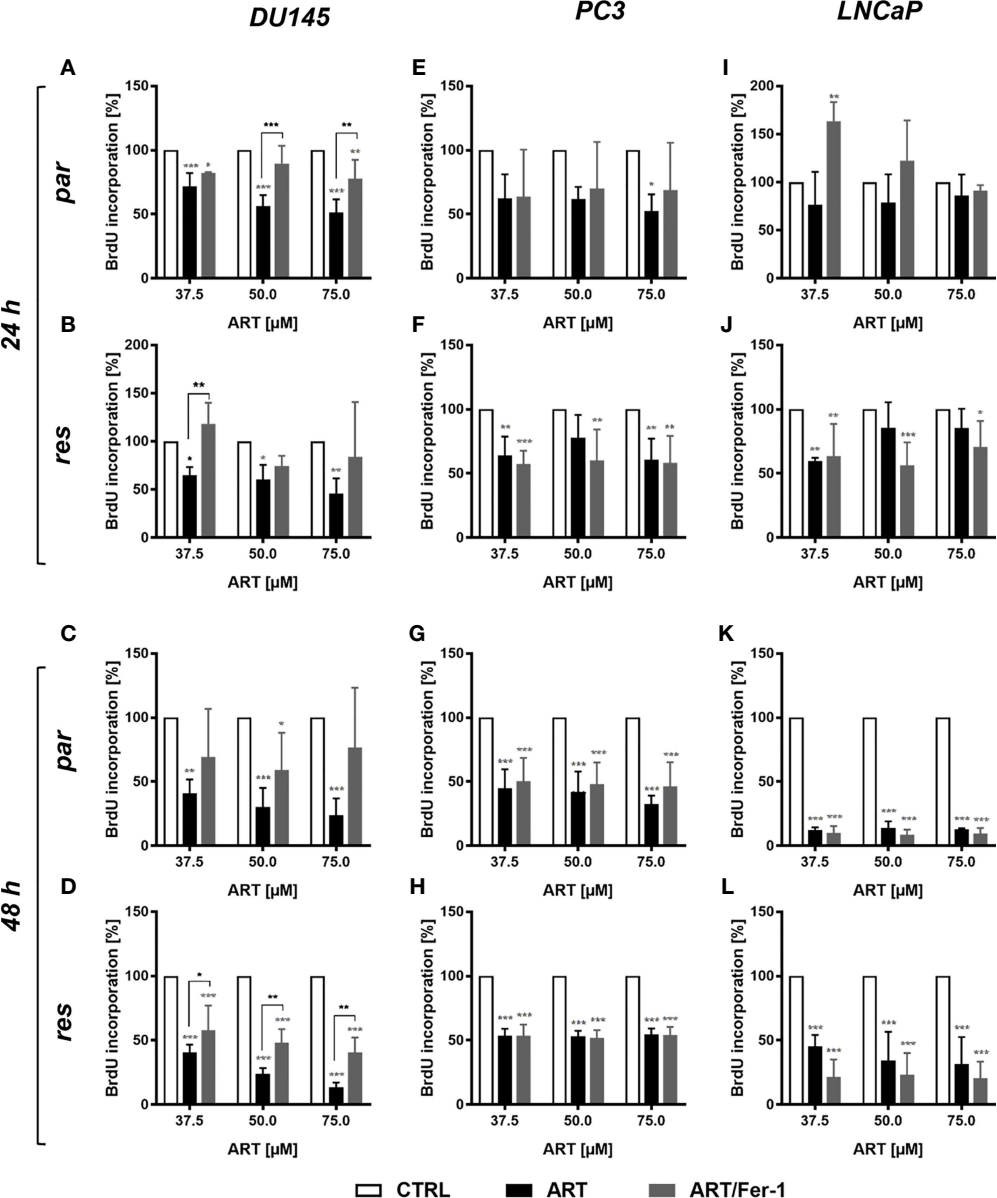
Ferroptosis induction: cell proliferation of parental (par) and DX-resistant (res) DU145, PC3, and LNCaP cells treated for 24 **(A, B, E, F, I, J)** and 48 h **(C, D, G, H, K, L)** with ART [37.5 - 75 µM], alone or in combination with ferrostatin-1 (Fer-1) [20 µM]. Untreated cells (100%) served as controls. Error bars indicate standard deviation (SD). Asterisk brackets indicate significant difference between ART and ART with ferrostatin-1 treatment. Significant difference compared to untreated controls indicated by: *p ≤ .05, **p ≤ .01, ***p ≤ .001. n = 3.

Neither cell cycle progression nor apoptosis was induced in the parental DU145 cells after ART treatment and could explain the observed growth and proliferation inhibition. Since ART has been shown to activate ferroptosis, an alternative iron-dependent cell death (29, 34, 35), ferroptosis was investigated. Combined application of ART with ferrostatin-1, a ferroptosis inhibitor, significantly counteracted ART’s inhibitory effect on proliferation in parental DU145 cells after 24 h (**Figure 7A**). This effect indicates that ferroptosis induction in parental DU145 cells contributes to the ART-mediated proliferation block. Also, in DX-resistant DU145 cells ferrostatin-1 application showed significant abrogation of ART’s [37.5 µM] anti-proliferative activity after 24 h, although to a lower extent (**Figure 7B**). The counteracting effect of ferrostatin-1 in the DX-resistant cells further increased after 48 h (**Figure 7D**), demonstrating a later activation of ferroptosis in DX-resistant DU145 cells. In contrast, in the parental DU145 cells the proliferation inhibition by ART was no longer significantly impaired, when combined with ferrostatin-1 for 48 h (**Figure 7C**), further corroborating the time-shift in ferroptosis-activation in parental and DX-resistant DU145 cells.

However, in parental and DX-resistant PC3 cells, combined ART application with ferrostatin-1 did not affect the efficacy of ART’s anti-proliferative activity **(** **Figures 7E–H**). Also in parental or DX-resistant LNCaP cells, additive administration of ferrostain-1 had no consequence on the ART-induced reduction of proliferation (**Figures 7I–L**). Thus, ART induces ferroptosis only in parental and DX-resistant DU145 cells. Therefore, only in these cell lines was the impact of ART on ROS production studied.

**Figure 8:**
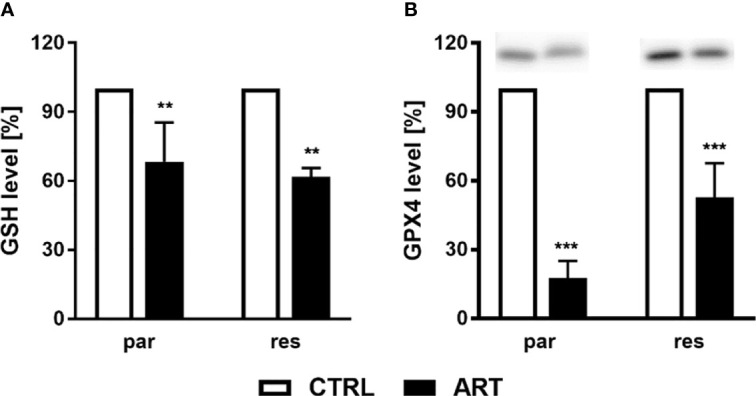
ROS production: GSH level [%] of DU145 cells after 24 h incubation with ART [37.5 µM], compared to untreated controls (set to 100%) **(A)**. n = 3. Pixel density analysis of the GPX4 expression after exposure to ART [37.5 µM] **(B)**. Error bars indicate standard deviation (SD). Significant difference to untreated controls indicated by: **p ≤.01, ***p ≤.001. n = 3. For detailed information regarding the Western blots, see **Figure S3**.

To investigate whether ART treatment results in ROS generation, the content of glutathione (GSH) and glutathione peroxidase 4 (GPX4), components of the antioxidant protection mechanism, were determined in ART-treated PCa cells. Biochemically, ferroptosis is characterized by consumption of extracellular GSH and decreased GPX4 levels. Thereby, reduction of GPX4 levels leads to intracellular accumulation of ROS, triggering ferroptosis. Administration of ART in parental and DX-resistant DU145 cells significantly reduced intracellular GSH content, compared to untreated controls, indicating higher GSH consumption and ROS generation (**Figure 8A**). Moreover, expression of GPX4 was significantly diminished in parental DU145 cells by 37.5 μM ART (**Figure 8B** and **Figure S3**). Significant down-regulation of GPX4 expression by ART was also observed in DX-resistant DU145 cells, albeit to a lesser extent than in the parental counterpart (**Figure 8B** and **Figure S3**).

## 4 Discussion

Despite recent progress, PCa remains a challenge without curative therapy for metastatic disease. Hence, the desire for complementary and alternative medicine (CAM) has gained widespread interest in the past decades. However, science-based knowledge concerning the anti-tumor properties of CAM is still limited. Therefore, the present study was designed to evaluate the therapeutic potential of ART in parental and DX-resistant PCa cell lines. Consistent with previous findings in therapy-sensitive PCa cells DU145, LNCaP (36), and 22rvl (19), a potent and dose-dependent inhibition of cell growth and proliferation by ART was observed. The ART concentrations affecting PCa cells *in vitro* are clinically relevant, since ART attaining these concentrations has been applied to treat patients with refractory solid tumors (37) and malaria (38). Besides the growth inhibitory effect in parental PCa cells, the current study revealed that ART also induces anti-growth and anti-proliferative activity in DX-resistant DU145, PC3, and LNCaP cells. Accordingly, ART disrupted resistance in other cancer types that have become insensitive to conventional therapeutic agents, including bicalutamide, an androgen receptor antagonist, applied formerly to treat metastatic castration-resistant prostate cancer (39). Recent studies have demonstrated ART-induced anti-cancer properties in parental and cisplatin-resistant bladder cancers (40) and sunitinib-resistant renal cell carcinomas (41). In line with these observations, ART also inhibited cell growth and proliferation in tongue (42), breast (43, 44), liver (45), colorectal (46) and esophageal (47) cancers. Moreover, the present investigation revealed that ART does not restrict the growth of ‘normal’, here primary non-tumor tubular epithelial, cells.

Reduced cell growth and proliferation of the DX-resistant DU145, PC3, and LNCaP and parental LNCaP cells were associated with G0/G1 phase arrest and a significant reduction of S phase (all) and G2/M phase cells (DU145). Consistent with this, ART-induced anti-proliferative activity was accompanied by an increase in the G0/G1 phase in human epidermoid carcinoma (48), endometrial cancer (49), bladder cancer (40) and renal carcinoma (41) cells. In good accordance with the G0/G1 phase arrest in the PCa cells, the cell cycle activating proteins CDK1, CDK2, cyclin A, and cyclin B, responsible for S phase and G2/M phase progression, were down-regulated, further corroborating the G0/G1 phase arrest. DNA replication in the S phase is controlled by the CDK2/cyclin A complex (50) and the activity of the cyclin B/CDK1 complex is required during the G2/M phase (51). Furthermore, expression of cyclin D1, which is involved in regulating G0/G1 phase progression (52), diminished after exposure to ART.

Apoptotic events occurred in parental PC3 and LNCaP cells as well as in the DX-resistant LNCaP and DU145 cells. In contrast, parental DU145 and DX-resistant PC3 cells did not display apoptotic cell death after 48 h exposure to ART. Thus, the mechanism behind the ART-induced inhibition of growth and proliferation in parental DU145 initially remained obscure. As a consequence, activation of alternative cell death signaling was postulated and evaluated as a possible explanation for these inhibitory effects of ART. Indeed, ferrostatin-1, a selective ferroptosis inhibitor, significantly abrogated the anti-proliferative activity of ART, exclusively in parental and DX-resistant DU145 cells, indicating ferroptosis induction. In good accordance with our data, it has been shown that ART efficiently induced ferroptosis in head and neck (28) as well as in pancreas (29) cancer cells. Furthermore, ART activated ferroptosis in hepatocellular carcinoma cells, when combined with sorafenib (53). In contrast, PC3 and LNCaP cells were not affected. In fact, only the DU145 cell line, among the chosen PCa cell lines, carries a mutation in the oncogenic KRAS gene, resulting in a gene fusion with the ubiquitin-conjugating enzyme UBE2L3 (54). Erastin, a specific ferroptosis inducer, caused preferential lethality in cells bearing a point mutation in the oncogenic HRAS(G12V) gene (55). Thus, the KRAS-UBE2L3 fusion protein might lead to ART-mediated induction of ferroptosis in DU145 cells. However, this is speculative and requires further investigation. In parental DU145 cells additional application of ferrostatin-1 already counteracted ART’s anti-proliferative impact after 24 h. A similar effect was evident in DX-resistant DU145 cells after 48 h, indicating a delayed initiation of ferroptosis in the DX-resistant DU145 cells, compared to the parental DU145 cells. This delay might explain the more potent proliferation inhibition after 48 h ART treatment in the parental, compared to the DX-resistant DU145 cells.

Several studies have indicated that ART induces DNA damage *via* oxidative stress and the generation of free radicals and reactive oxygen species (56, 57). A main feature of ferroptosis is lipid peroxidation of the cellular and organelle membranes, due to elevated intracellular ROS, causing oxidative cell death (58–60). Ferroptosis-mediated ROS is known to be accompanied by a depletion of GSH and the blockade of the antioxidant defense mechanisms with the Systems-xc and GSH-dependent peroxidase, GPX4 (58–60). In good accordance with this, in both parental and DX-resistant DU145 cells significantly reduced GSH levels were apparent after exposure to ART, confirming ROS formation under ART administration. Similarly, ART increased ROS and decreased GSH in the most aggressive triple-negative breast cancer cells, resulting in intracellular oxidative imbalance and ferroptosis (61). Furthermore, ART induced ferroptosis in head and neck cancer cells by decreasing cellular GSH levels and increasing lipid ROS levels (28). This effect could be blocked by co-incubation with ferrostatin-1. GPX4 catalyzes the reduction of GSH to oxidized GSSG, using GSH as an essential cofactor, thereby reducing ROS to H_2_O (62). Silencing GPX4 by shRNA, which leads to a partial knockdown of GPX4, sensitized fibrosarcoma and renal carcinoma cells to a ferroptosis-induced lethality by erastin (63).Consistent with this, the current study revealed a significant decrease of GPX4 expression in both parental and DX-resistant cells after ART treatment, indicating inhibited GPX4 activity accompanied by absent GSH regeneration and therewith accumulation of ROS.

Since the antitumor effects of ART were apparent in both androgen-sensitive (LNCaP) and androgen-insensitive PCa-cells (PC3 & DU-145), ART’s action seems to be androgen receptor-independent. However, an impact on androgen receptors cannot be totally excluded, as its expression was not evaluated. Other investigators have revealed a reduction in androgen receptors after ART treatment in androgen receptor-positive 22rvl cells (19).

## 5 Conclusions

ART exhibited tumor suppressive potential in regard to the progressive growth of parental and therapy-resistant PCa cells. Cell-type-specific modes of action were induced by ART, leading to cell cycle arrest, apoptosis and/or ferroptosis. These actions were accompanied by impaired expression of cell cycle activating proteins, mainly CDK1, CDK2, cyclin A, cyclin B, and cyclin D1, modulated expression of apoptotic proteins, and an increase in ROS. Therefore, ART may hold promise as a complementary therapeutic option in patients with advanced and even therapy-resistant PCa.

## Data Availability Statement

The original contributions presented in the study are included in the article/**Supplementary Material**. Further inquiries can be directed to the corresponding author.

## Author Contributions

Conceptualization, EJ. Methodology, VB, OV, SM, HE, PS, PB, KS, AT, MP, ZC, JC, and MM. Software, SM. Validation, OV and SM. Formal analysis, VB and OV. Investigation, EJ and OV. Resources, EJ, PB, TE, MP, ZC, JC, and MM. Data curation, OV. Writing—original draft preparation, OV. Writing—review and editing, EJ, HE, IT, AH, TE, and MM. Visualization, OV. Supervision, EJ. Project administration, EJ. Funding acquisition, EJ, MM, and JC. All authors have read and agreed to the published version of the manuscript.

## Funding

This research was funded by the Brigitta und Norbert Muth Stiftung, grant number 01/2018 (EJ), Hilfe für krebskranke Kinder Frankfurt e.V. (JCJr.), Frankfurter Stiftung für krebskranke Kinder (JCJr), and the Kent Cancer Trust (MM).

## Conflict of Interest

The authors declare that the research was conducted in the absence of any commercial or financial relationships that could be construed as a potential conflict of interest.

## Publisher’s Note

All claims expressed in this article are solely those of the authors and do not necessarily represent those of their affiliated organizations, or those of the publisher, the editors and the reviewers. Any product that may be evaluated in this article, or claim that may be made by its manufacturer, is not guaranteed or endorsed by the publisher.
