# Supplementary material for: Artesunate Inhibits the Growth Behavior of Docetaxel-Resistant Prostate Cancer Cells
Source: Front Oncol. 2022 Feb 7;12:789284. doi: 10.3389/fonc.2022.789284 (PMC8859178; doi:10.3389/fonc.2022.789284)
Supplement: Supplementary file 1 [file DataSheet_1.pdf]

Cell cycle regulating proteins

Figure S1.1 (A) DU145par and DU145res CDK1 (34kDa)
(B) Coomassie Brilliant Blue

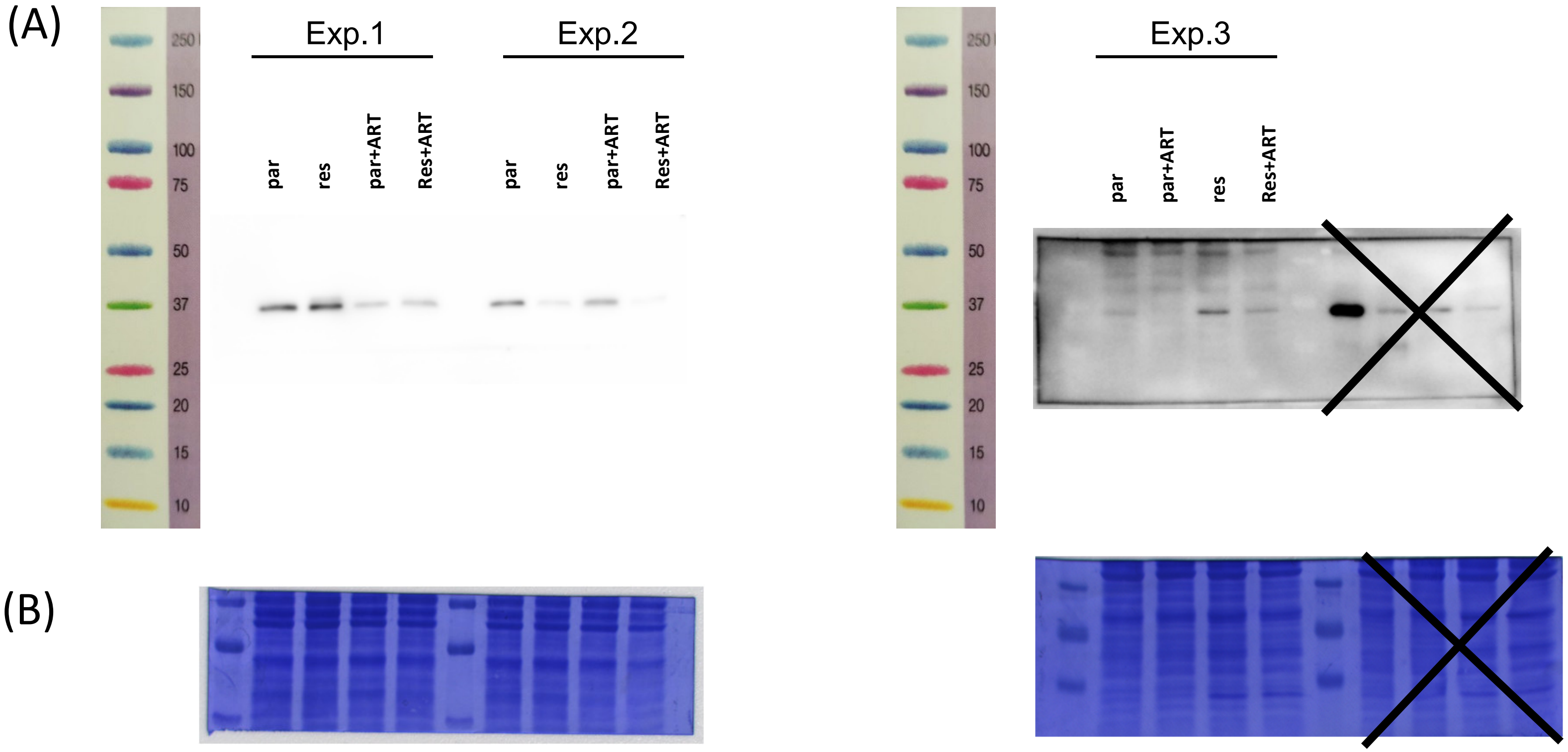

Figure S1.1: Detailed information about Figure 4 - Protein expression profile of cell cycle regulating proteins in parental and resistant DU145 cells. Protein expression of CDK1 (A), corresponding Coomassie blue staining of total protein (B).

Figure S1.2 (A) DU145par and DU145res CDK2 (33 kDa)
(B) Coomassie Brilliant Blue

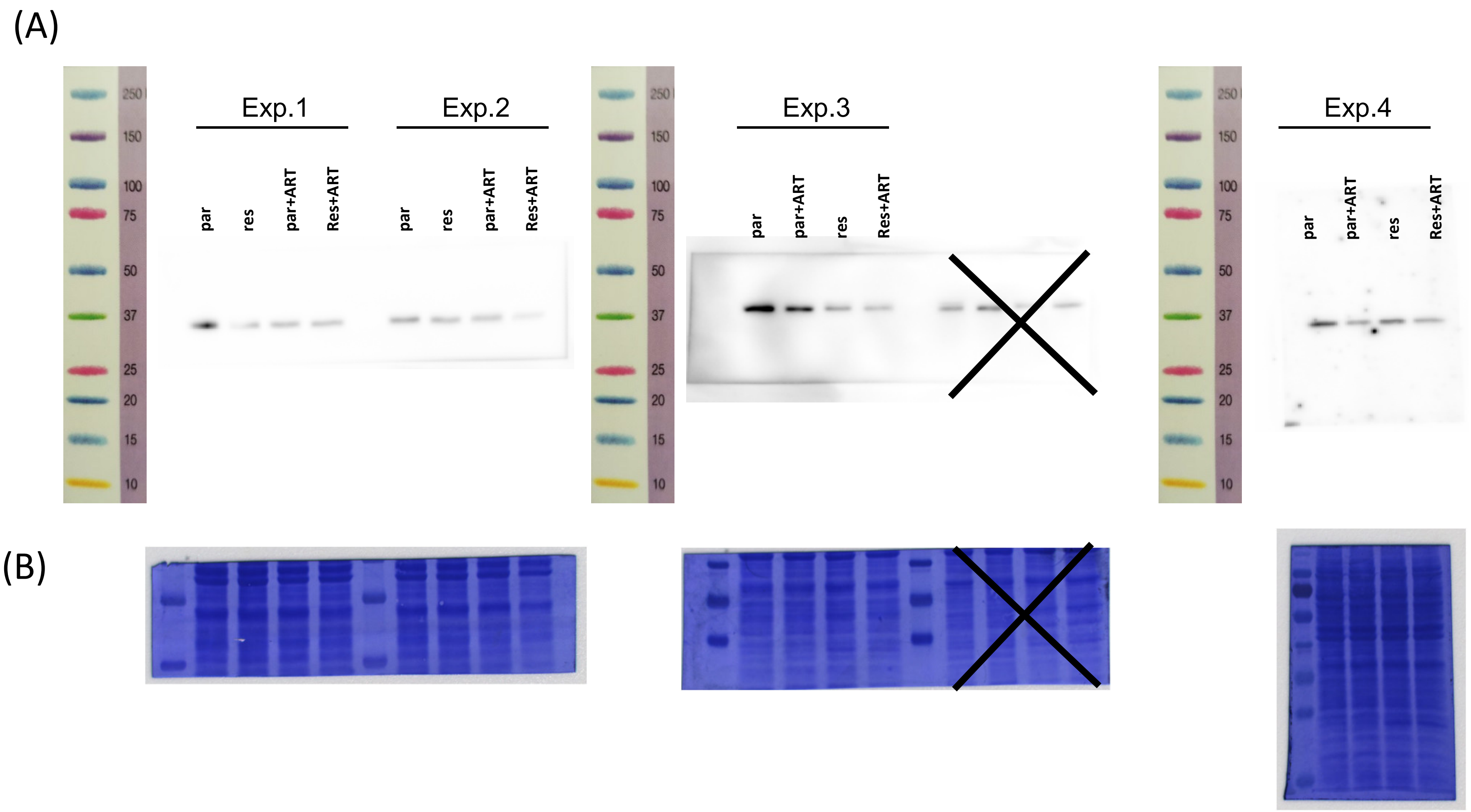

Figure S1.2: Detailed information about Figure 4 - Protein expression profile of cell cycle regulating proteins in parental and resistant DU145 cells. Protein expression of CDK2 (A), corresponding Coomassie blue staining of total protein (B).

Figure S1.3 (A) DU145par and DU145res Cyclin A (60 kDa)
(B) Coomassie Brilliant Blue

(A)

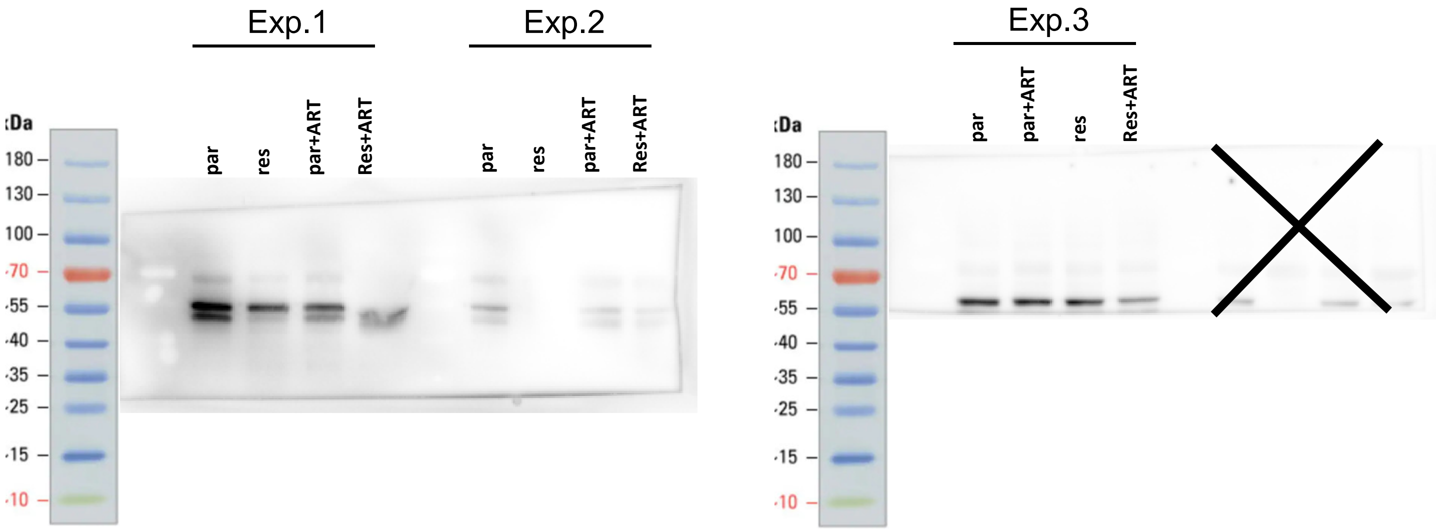

(B)

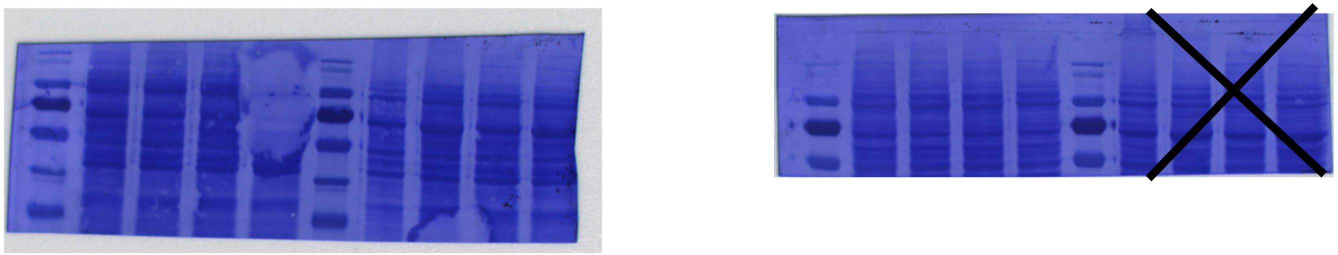

Figure S1.3: Detailed information about Figure 4 - Protein expression profile of cell cycle regulating proteins in parental and resistant DU145 cells. Protein expression of Cyclin A (A), corresponding Coomassie blue staining of total protein (B).

Figure S1.4 (A) DU145par and DU145res Cyclin B (62 kDa)
(B) Coomassie Brilliant Blue

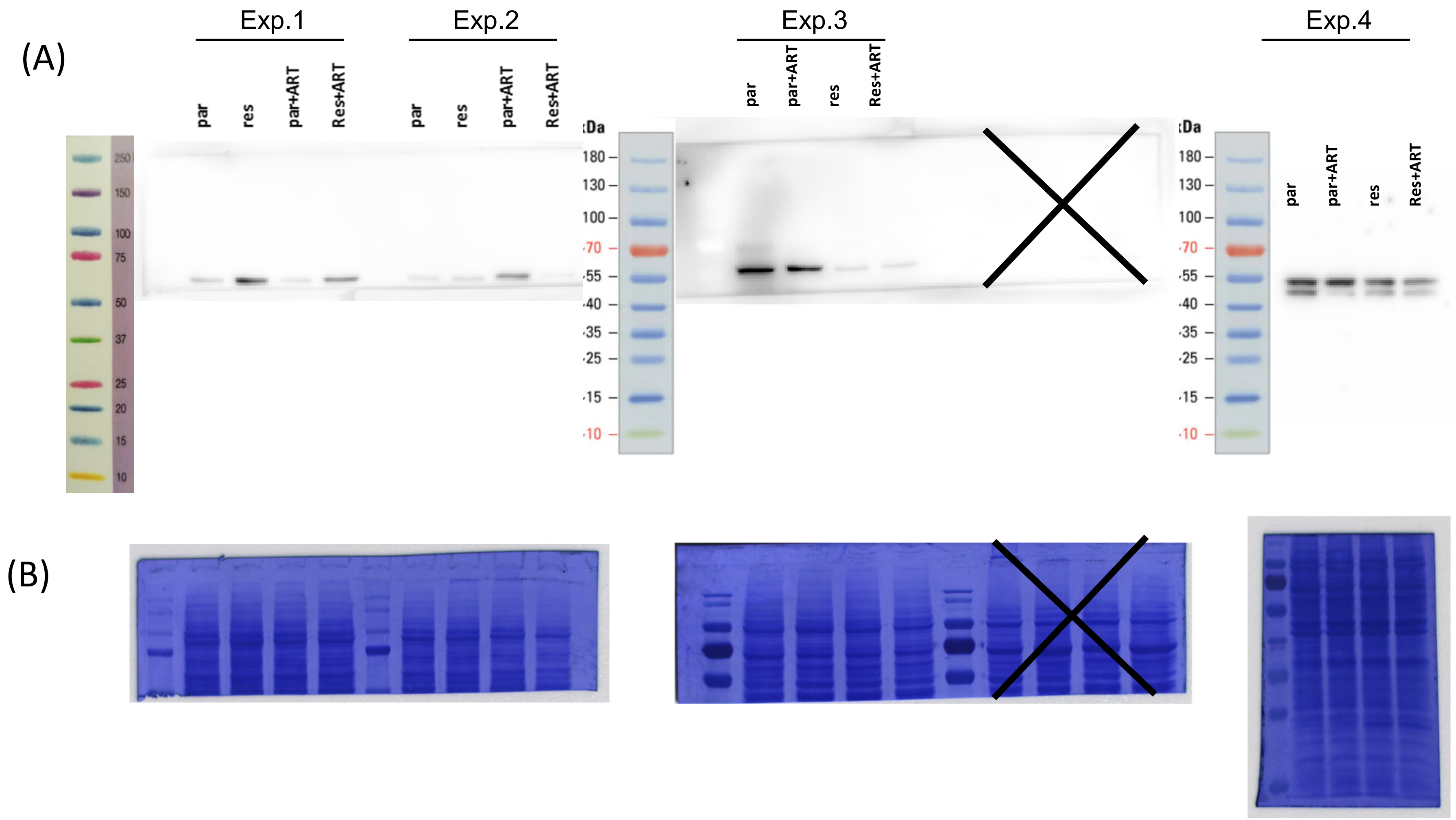

Figure S1.4: Detailed information about Figure 4 - Protein expression profile of cell cycle regulating proteins in parental and resistant DU145 cells. Protein expression of Cyclin B (A), corresponding Coomassie blue staining of total protein (B).

Figure S1.5 (A) DU145par and DU145res Cyclin D1 (34 kDa)
(B) Coomassie Brilliant Blue

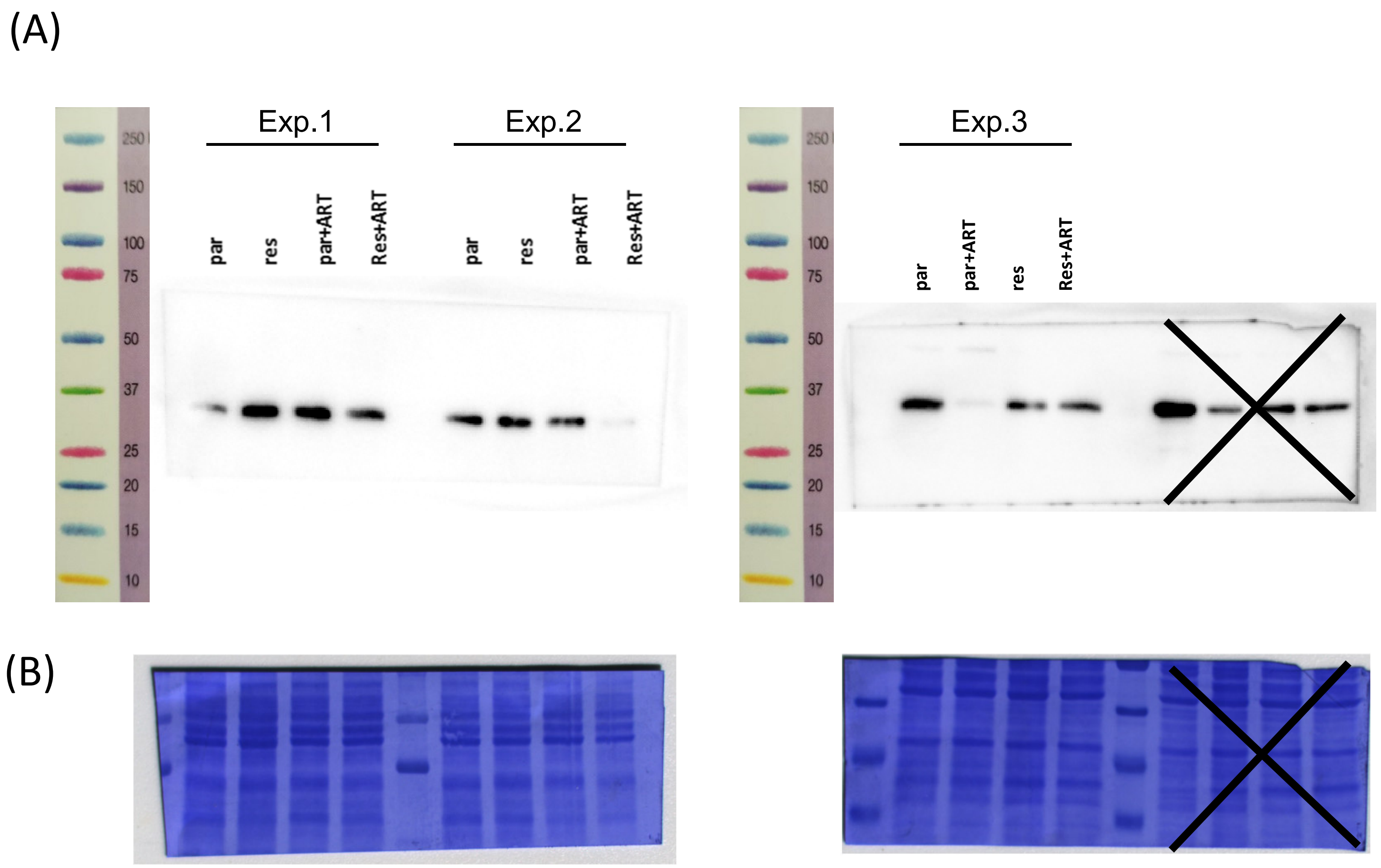

Figure S1.5: Detailed information about Figure 4 - Protein expression profile of cell cycle regulating proteins in parental and resistant DU145 cells. Protein expression of Cyclin D1 (A), corresponding Coomassie blue staining of total protein (B).

Figure S1.6 (A) PC3par and PC3res CDK1 (34kDa)
(B) Coomassie Brilliant Blue

(A)

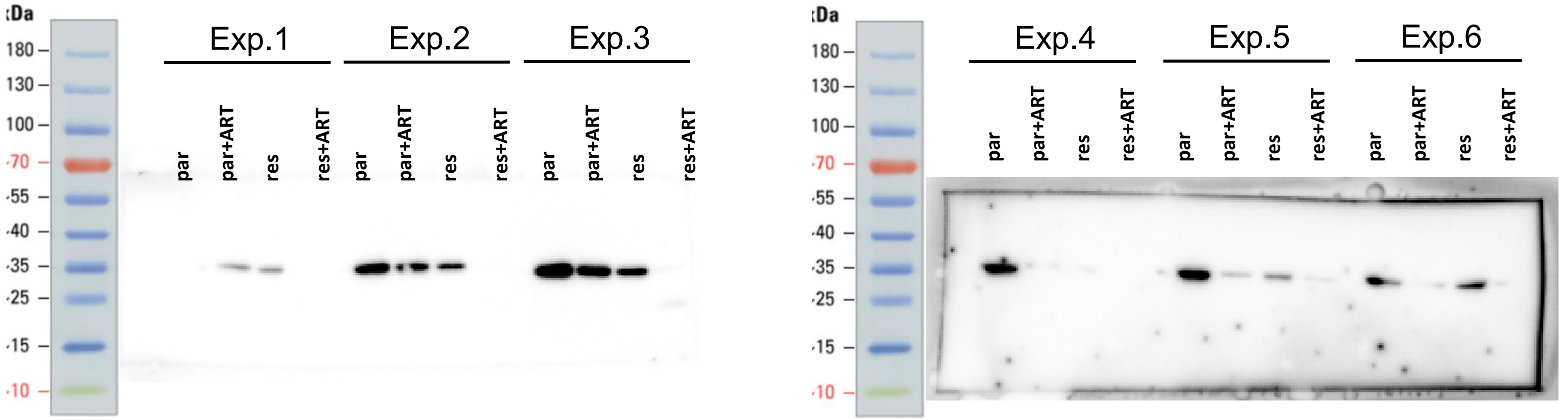

(B)

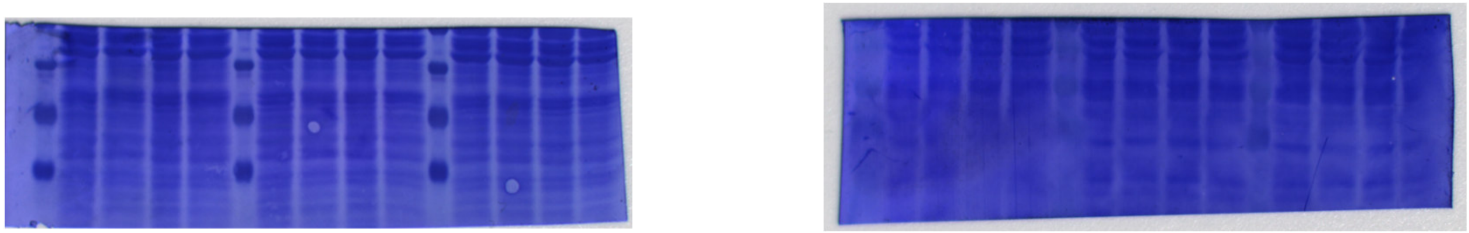

Figure S1.6: Detailed information about Figure 4 - Protein expression profile of cell cycle regulating proteins in parental and resistant PC3 cells. Protein expression of CDK1 (A), corresponding Coomassie blue staining of total protein (B).

Figure S1.7 (A) PC3par and PC3res CDK2 (33kDa)
(B) Coomassie Brilliant Blue

(A)

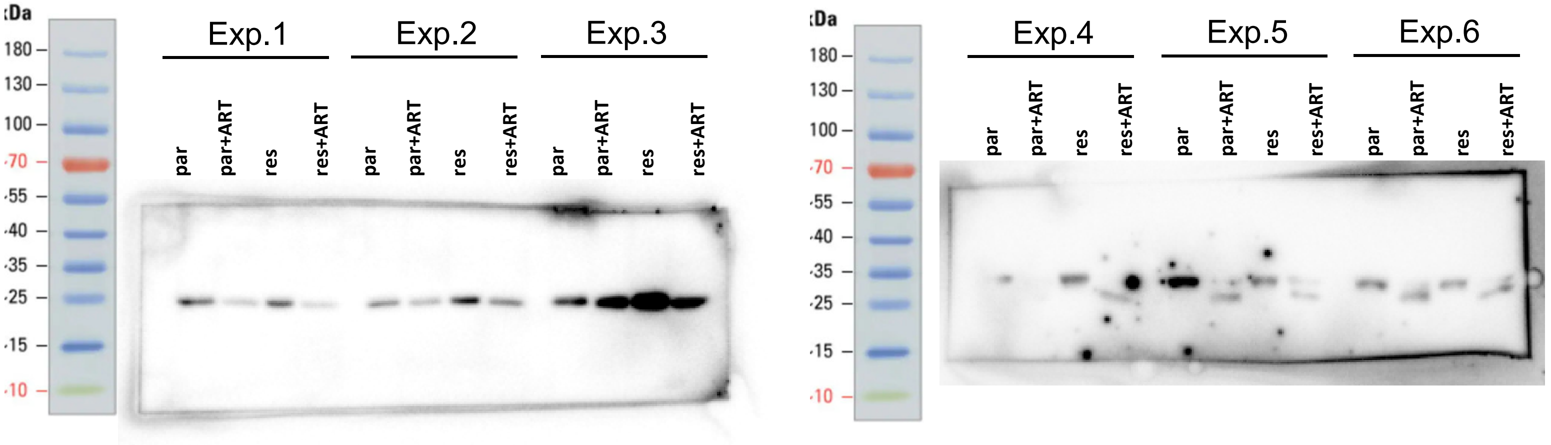

(B)

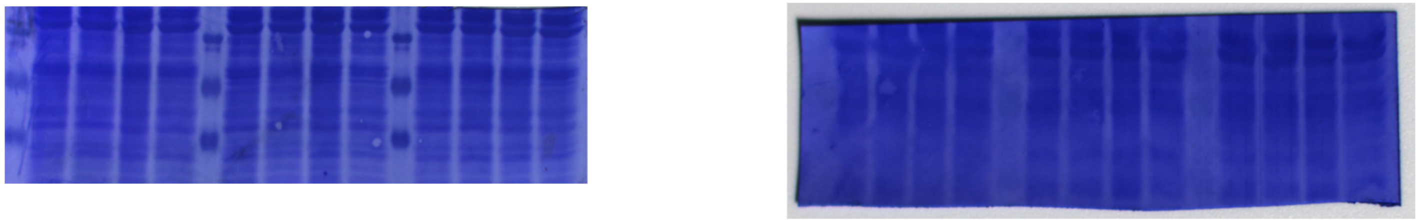

Figure S1.7: Detailed information about Figure 4 - Protein expression profile of cell cycle regulating proteins in parental and resistant PC3 cells. Protein expression of CDK2 (A), corresponding Coomassie blue staining of total protein (B).

Figure S1.8 (A) PC3par and PC3res Cyclin A (60 kDa)
(B) Coomassie Brilliant Blue

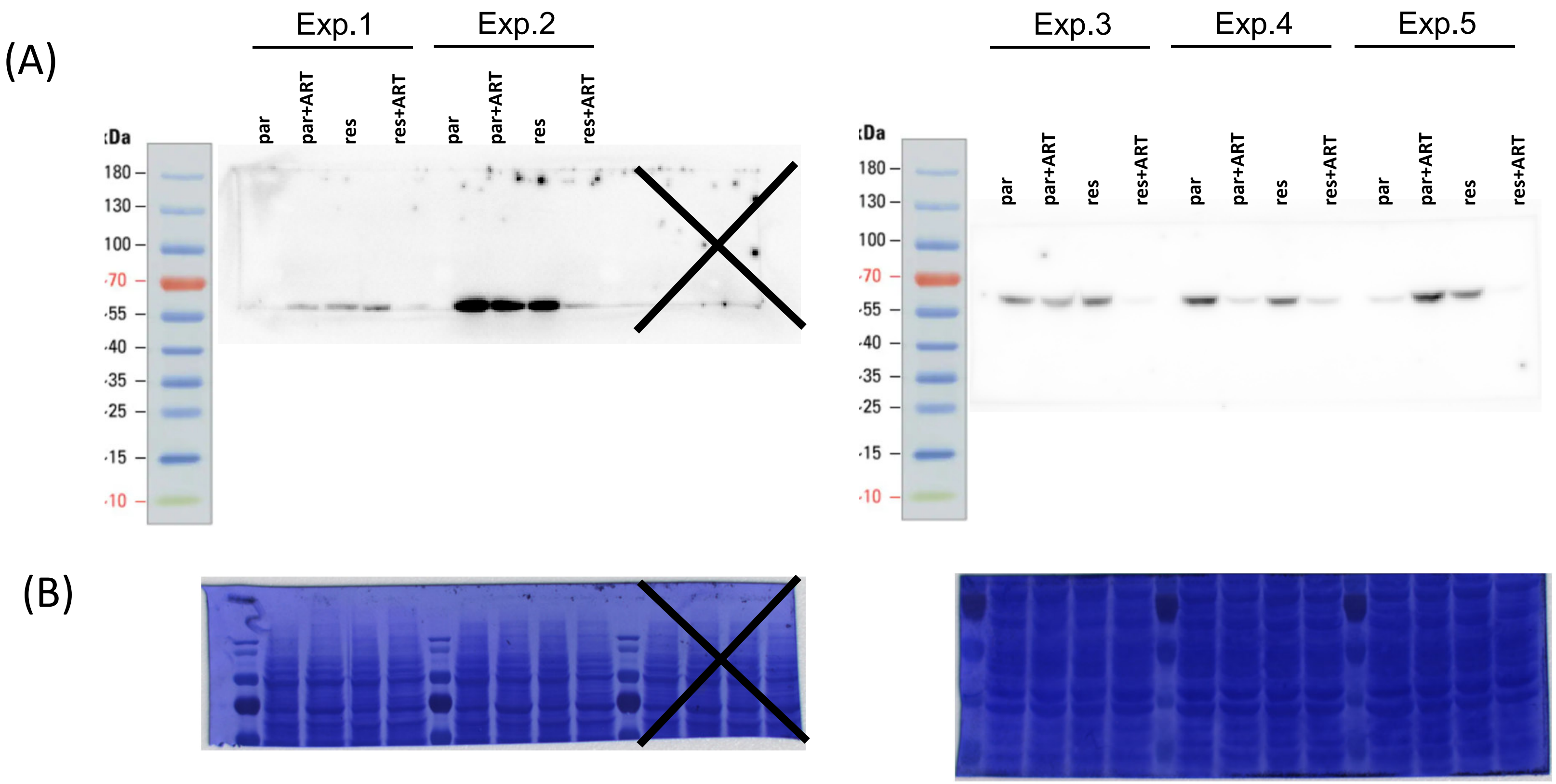

Figure S1.8: Detailed information about Figure 4 - Protein expression profile of cell cycle regulating proteins in parental and resistant PC3 cells. Protein expression of Cyclin A (A), corresponding Coomassie blue staining of total protein (B).

Figure S1.9 (A) PC3par and PC3res Cyclin B (62 kDa)
(B) Coomassie Brilliant Blue

(A)

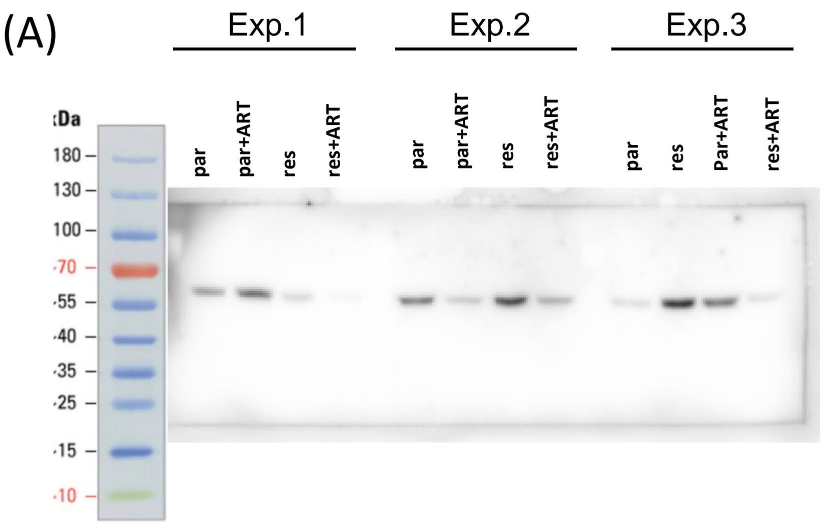

(B)

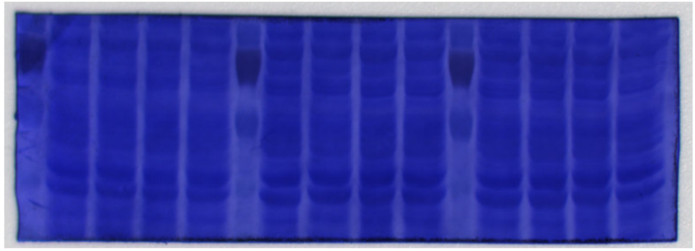

Figure S1.9: Detailed information about Figure 4 - Protein expression profile of cell cycle regulating proteins in parental and resistant PC3 cells. Protein expression of Cyclin B (A), corresponding Coomassie blue staining of total protein (B).

Figure S1.10 (A) PC3par and PC3res Cyclin D1 (34 kDa)
(B) Coomassie Brilliant Blue

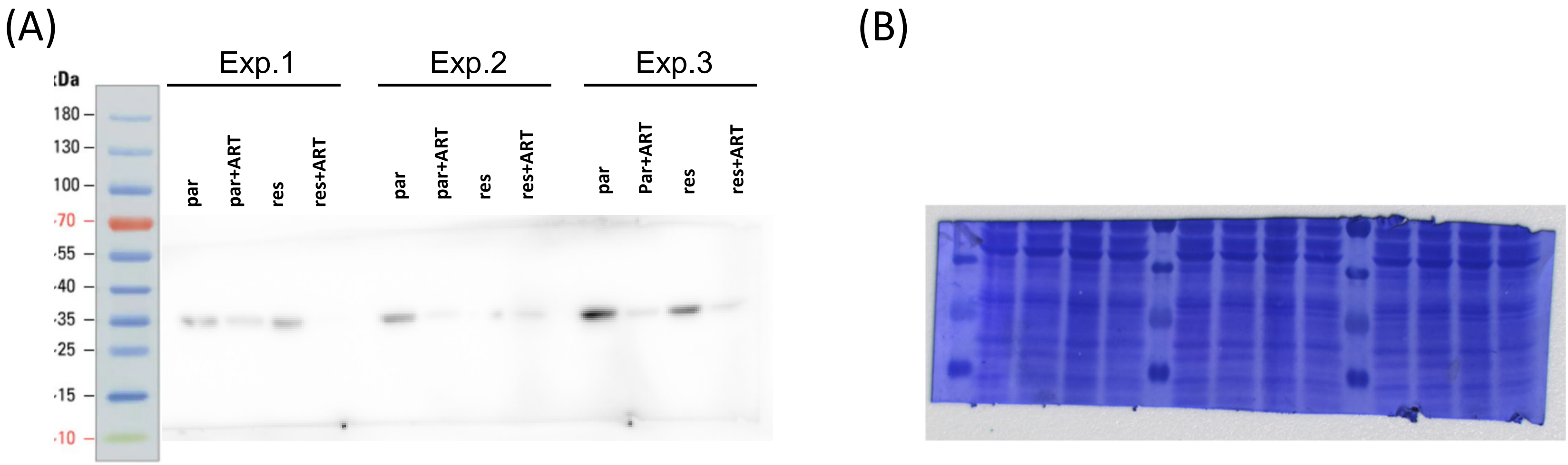

Figure S1.10: Detailed information about Figure 4 - Protein expression profile of cell cycle regulating proteins in parental and resistant PC3 cells. Protein expression of Cyclin D1 (A), corresponding Coomassie blue staining of total protein (B).

Figure S1.11 (A) LNCaPpar and LNCaPres CDK1 (34kDa)
(B) Coomassie Brilliant Blue

(A)

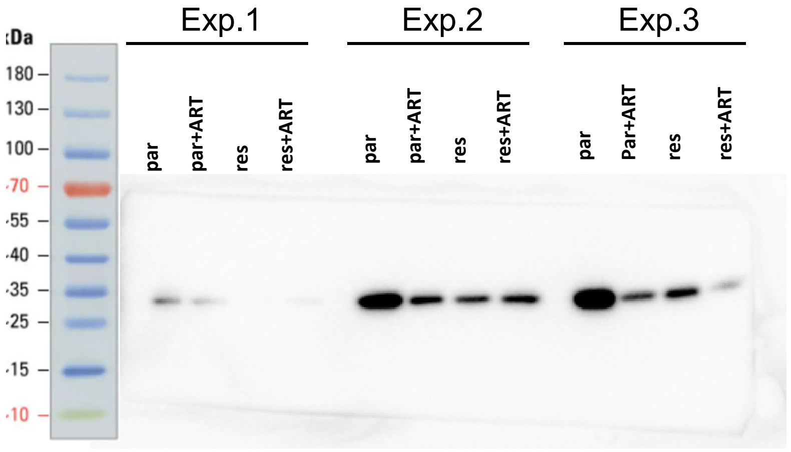

(B)

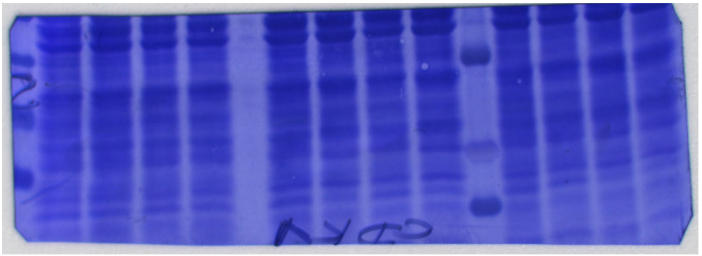

Figure S1.11: Detailed information about Figure 4 - Protein expression profile of cell cycle regulating proteins in parental and resistant LNCaP cells. Protein expression of CDK1 (A), corresponding Coomassie blue staining of total protein (B).

Figure S1.12 (A) LNCaPpar and LNCaPres CDK2 (33 kDa)
(B) Coomassie Brilliant Blue

(A)

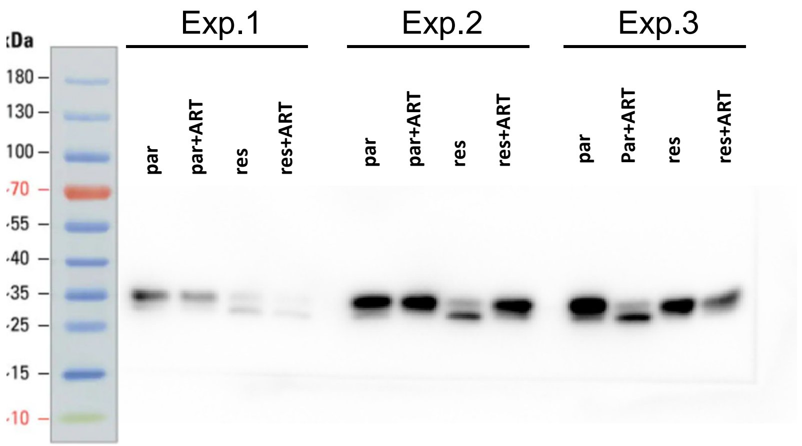

(B)

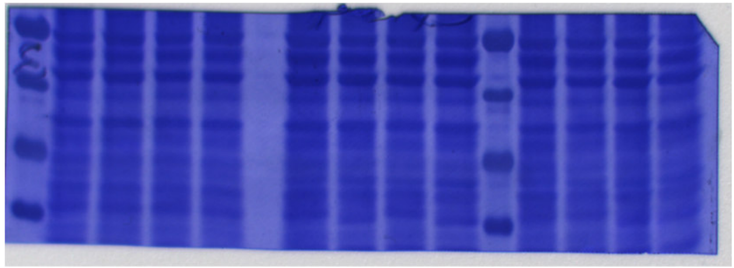

Figure S1.12: Detailed information about Figure 4 - Protein expression profile of cell cycle regulating proteins in parental and resistant LNCaP cells. Protein expression of CDK2 (A), corresponding Coomassie blue staining of total protein (B).

Figure S1.13 (A) LNCaPpar and LNCaPres Cyclin A (60 kDa)

(B) Coomassie Brilliant Blue

(A) (B)

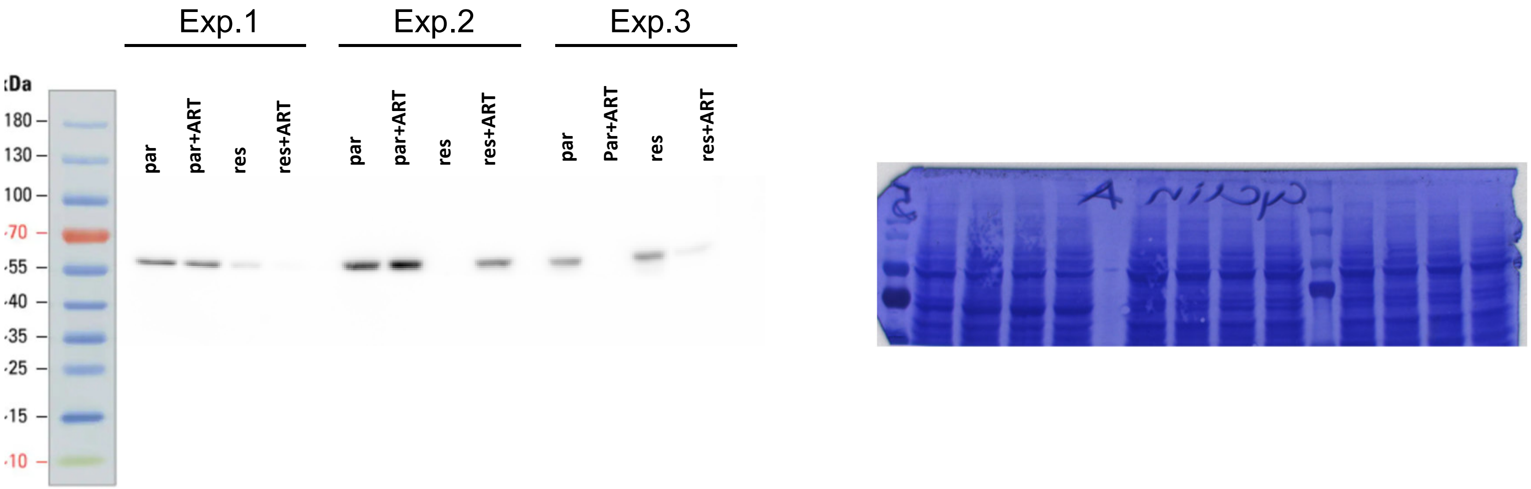

Figure S1.13: Detailed information about Figure 4 - Protein expression profile of cell cycle regulating proteins in parental and resistant LNCaP cells. Protein expression of Cyclin A (A), corresponding Coomassie blue staining of total protein (B).

Figure S1.14 (A) LNCaPpar and LNCaPres Cyclin B (62 kDa)
(B) Coomassie Brilliant Blue

(A)

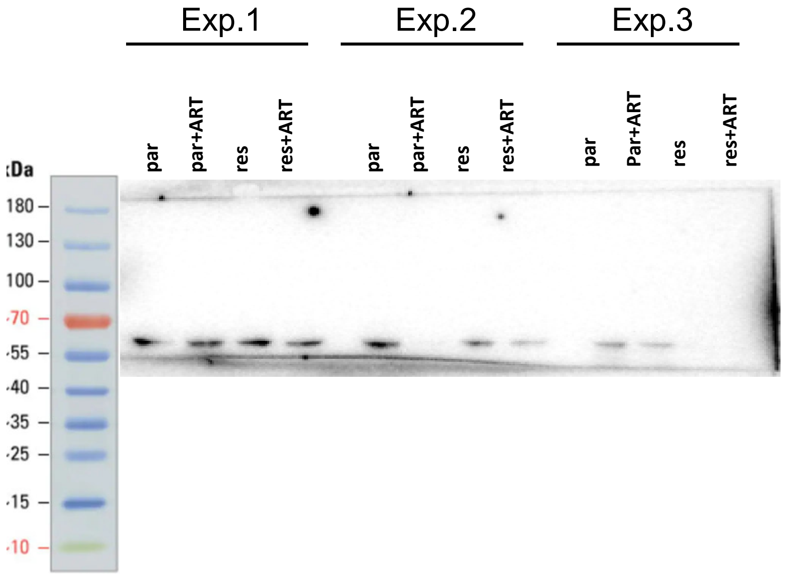

(B)

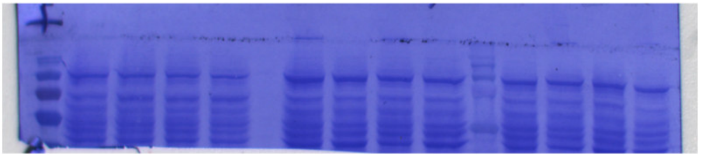

Figure S1.14: Detailed information about Figure 4 - Protein expression profile of cell cycle regulating proteins in parental and resistant LNCaP cells. Protein expression of Cyclin B (A), corresponding Coomassie blue staining of total protein (B).

Figure S1.15 (A) LNCaPpar and LNCaPres Cyclin D1 (34 kDa)
(B) Coomassie Brilliant Blue

(A)

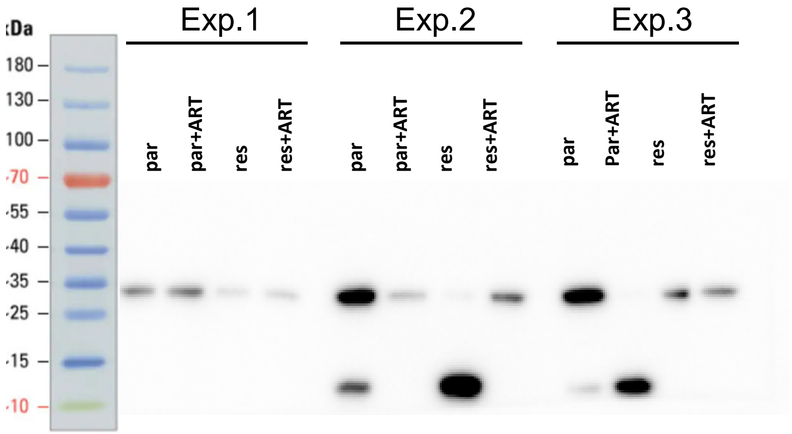

(B)

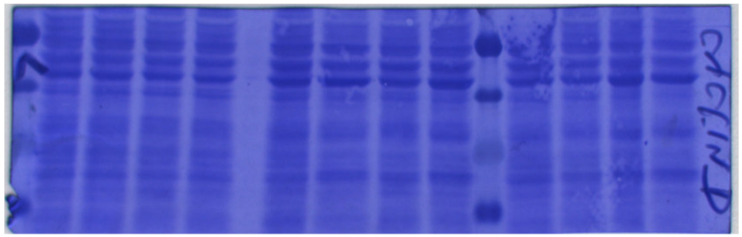

Figure S1.15: Detailed information about Figure 4 - Protein expression profile of cell cycle regulating proteins in parental and resistant LNCaP cells. Protein expression of Cyclin D1 (A), corresponding Coomassie blue staining of total protein (B).

Apoptosis related proteins

Figure S2.1 (A) DU145par and DU145res, caspase 3 (35 kDa)
(B) Coomassie Brilliant Blue

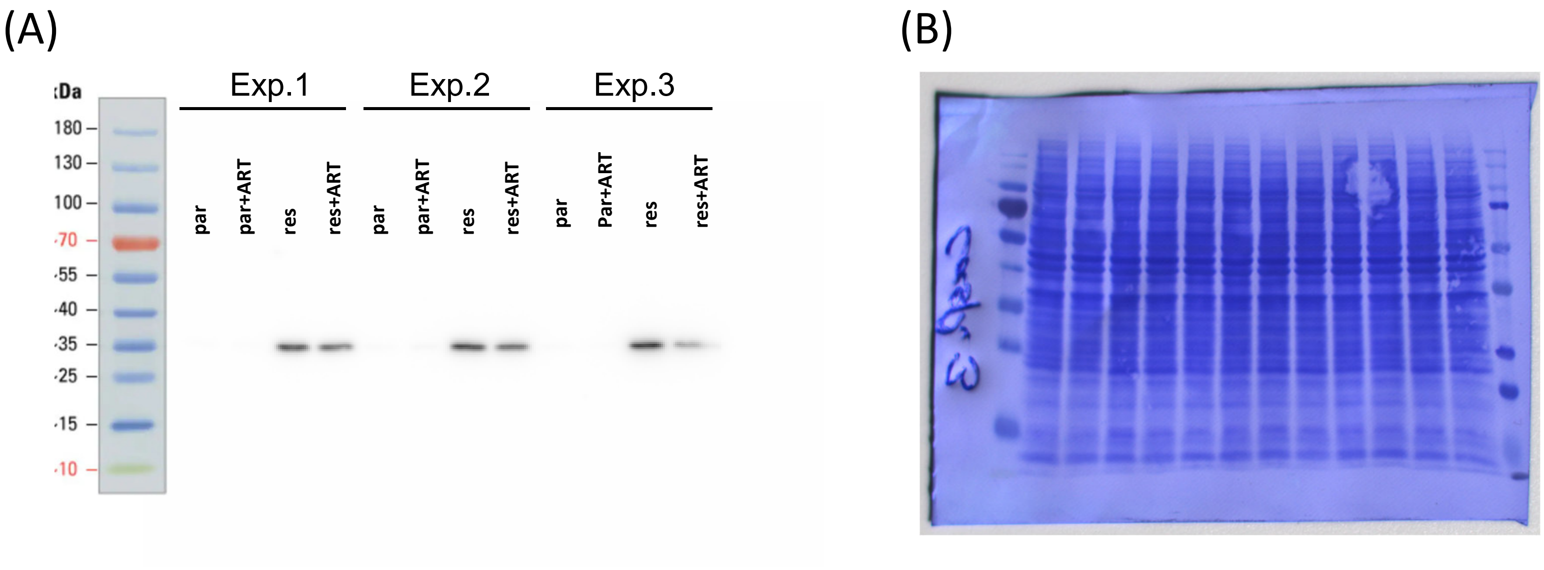

Figure S2.1: Detailed information about Figure 6 - Protein expression profile of DNA damage repair and apoptosis associated proteins in parental and resistant DU145 cells. Protein expression of caspase 3 (A), corresponding Coomassie blue staining of total protein (B).

Figure S2.2 (A) DU145par and DU145res, caspase 8 (57 kDa)
(B) Coomassie Brilliant Blue

(A)

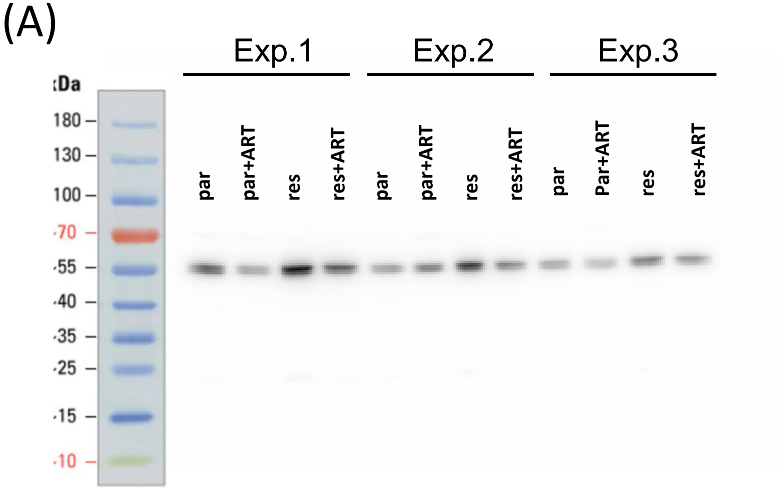

(B)

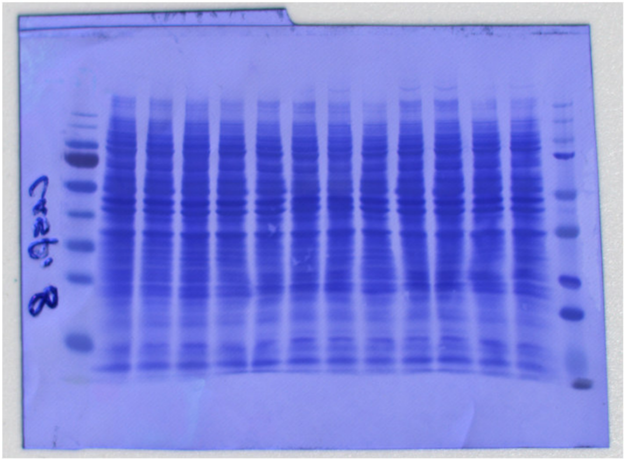

Figure S2.2: Detailed information about Figure 6 - Protein expression profile of DNA damage repair and apoptosis associated proteins in parental and resistant DU145 cells. Protein expression of caspase 8 (A), corresponding Coomassie blue staining of total protein (B).

Figure S2.3 (A) DU145par and DU145res PARP-1 (116 kDa)
(B) Coomassie Brilliant Blue

(A)

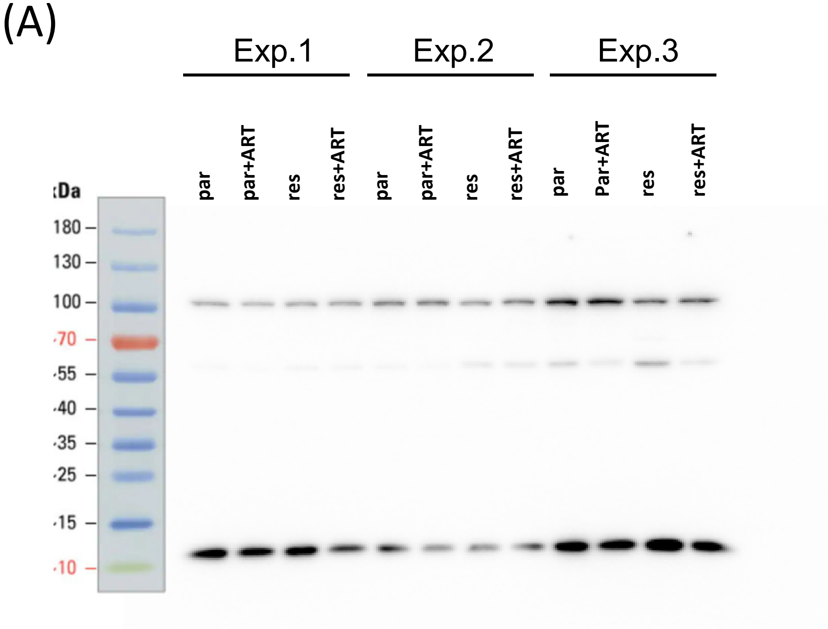

(B)

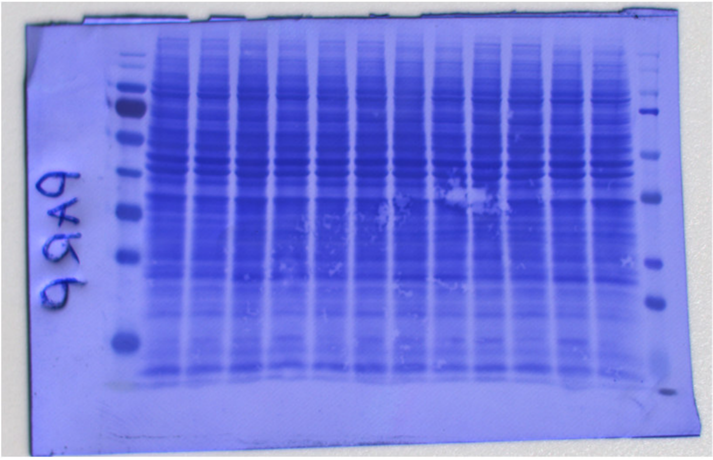

Figure S2.3: Detailed information about Figure 6 - Protein expression profile of DNA damage repair and apoptosis associated proteins in parental and resistant DU145 cells. Protein expression of PARP-1 (A), corresponding Coomassie blue staining of total protein (B).

Figure S2.4 (A) PC3par and PC3res, caspase 3 (35 kDa)
(B) Coomassie Brilliant Blue

(A)

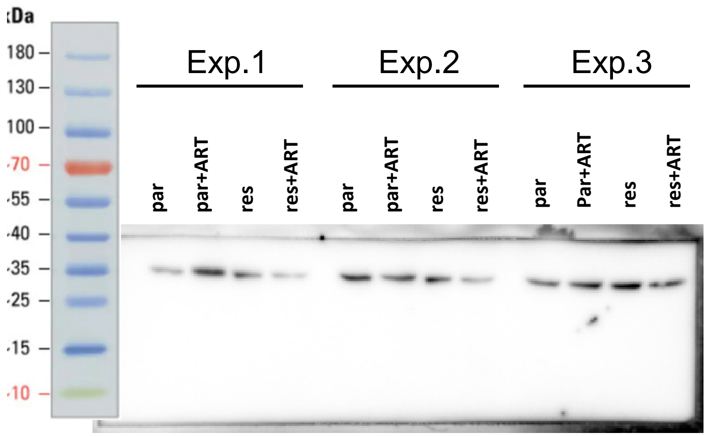

(B)

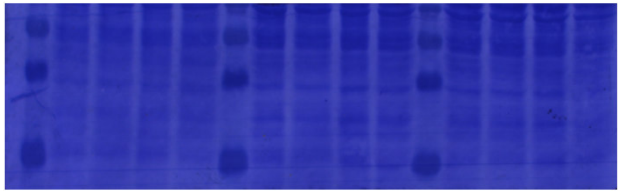

Figure S2.4: Detailed information about Figure 6 - Protein expression profile of DNA damage repair and apoptosis associated proteins in parental and resistant PC3 cells. Protein expression of caspase 3 (A), corresponding Coomassie blue staining of total protein (B).

Figure S2.5 (A) PC3par and PC3res, caspase 8 (57 kDa)
(B) Coomassie Brilliant Blue

(A)

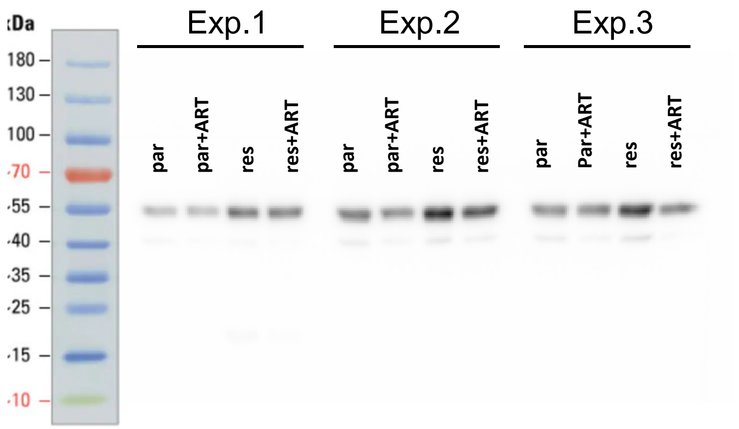

(B)

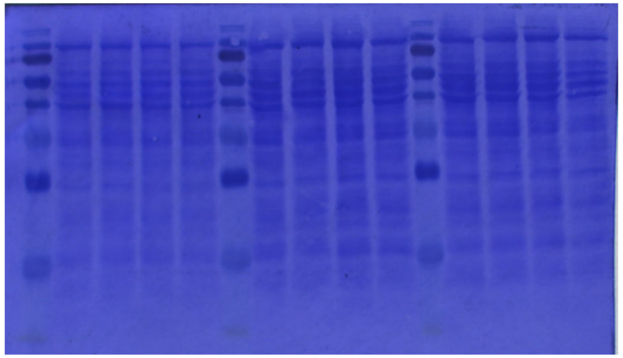

Figure S2.5: Detailed information about Figure 6 - Protein expression profile of DNA damage repair and apoptosis associated proteins in parental and resistant PC3 cells. Protein expression of caspase 8 (A), corresponding Coomassie blue staining of total protein (B).

Figure S2.6 (A) PC3par and PC3res PARP-1 (116 kDa)
(B) Coomassie Brilliant Blue

(A)

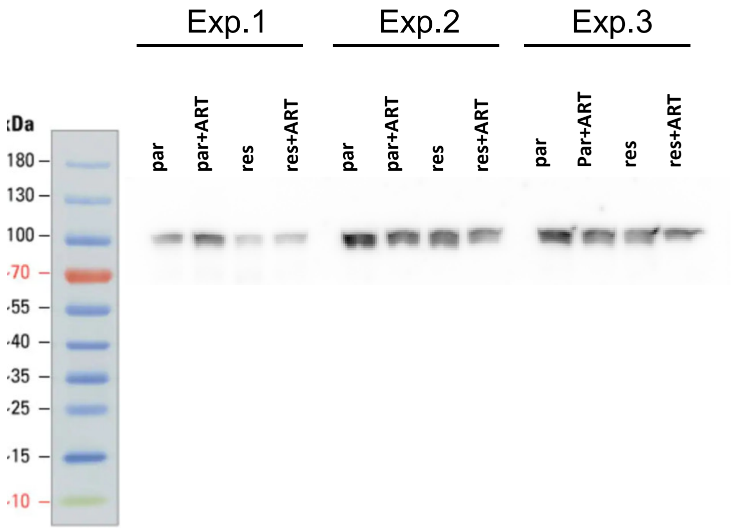

(B)

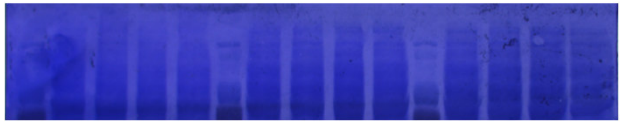

Figure S2.6: Detailed information about Figure 6 - Protein expression profile of DNA damage repair and apoptosis associated proteins in parental and resistant PC3 cells. Protein expression of PARP-1 (A), corresponding Coomassie blue staining of total protein (B).

Figure S2.7 (A) LNCaPpar and LNCaPres, caspase 3 (35 kDa)
(B) Coomassie Brilliant Blue

(A)

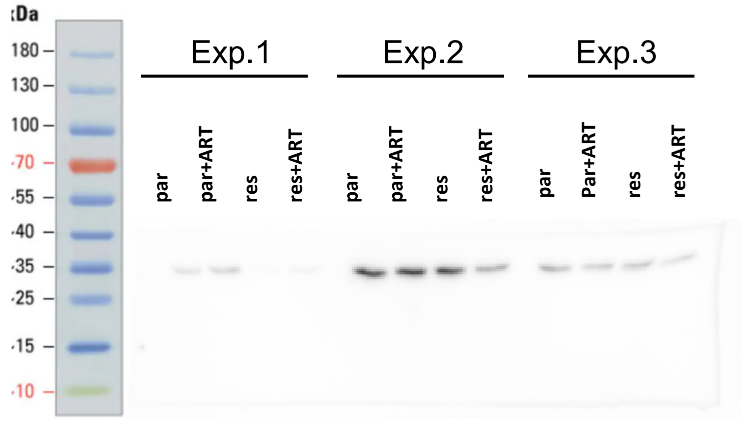

(B)

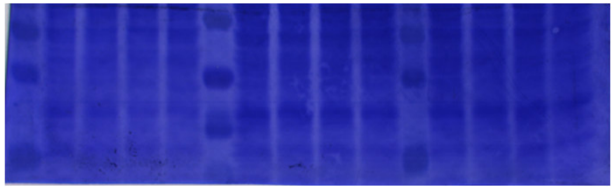

Figure S2.7: Detailed information about Figure 6 - Protein expression profile of DNA damage repair and apoptosis associated proteins in parental and resistant LNCaP cells. Protein expression of caspase 3 (A), corresponding Coomassie blue staining of total protein (B).

Figure S2.8 (A) LNCaPpar and LNCaPres, caspase 8 (57 kDa)
(B) Coomassie Brilliant Blue

(A)

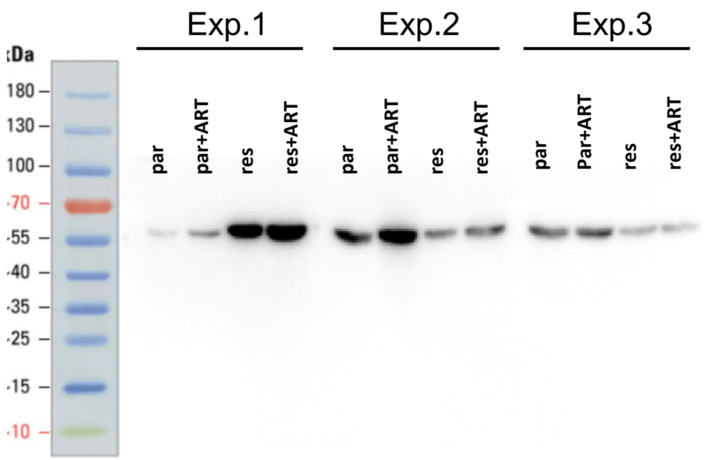

(B)

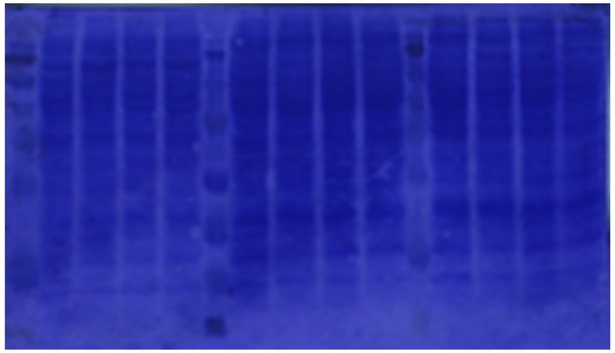

Figure S2.8: Detailed information about Figure 6 - Protein expression profile of DNA damage repair and apoptosis associated proteins in parental and resistant LNCaP cells. Protein expression of caspase 8 (A), corresponding Coomassie blue staining of total protein (B).

Figure S2.9 (A) LNCaPpar and LNCaPres PARP-1 (116 kDa)
(B) Coomassie Brilliant Blue

(A)

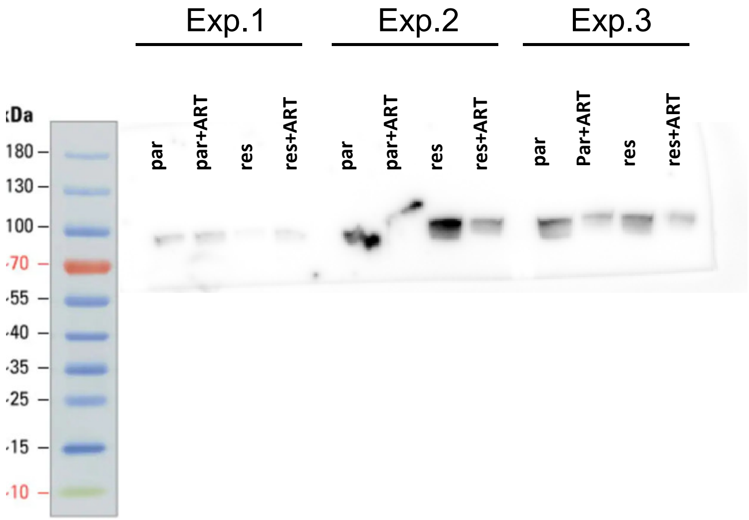

(B)

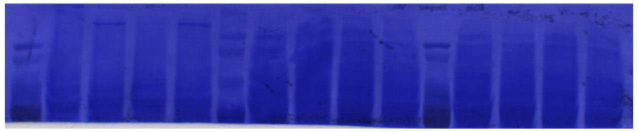

Figure S2.9: Detailed information about Figure 6 - Protein expression profile of DNA damage repair and apoptosis associated proteins in parental and resistant LNCaP cells. Protein expression of PARP-1 (A), corresponding Coomassie blue staining of total protein (B).

Ferroptosis related protein

Figure S3 (A) DU145par and DU145res, GPX4 (24 kDa)
(B) Coomassie Brilliant Blue

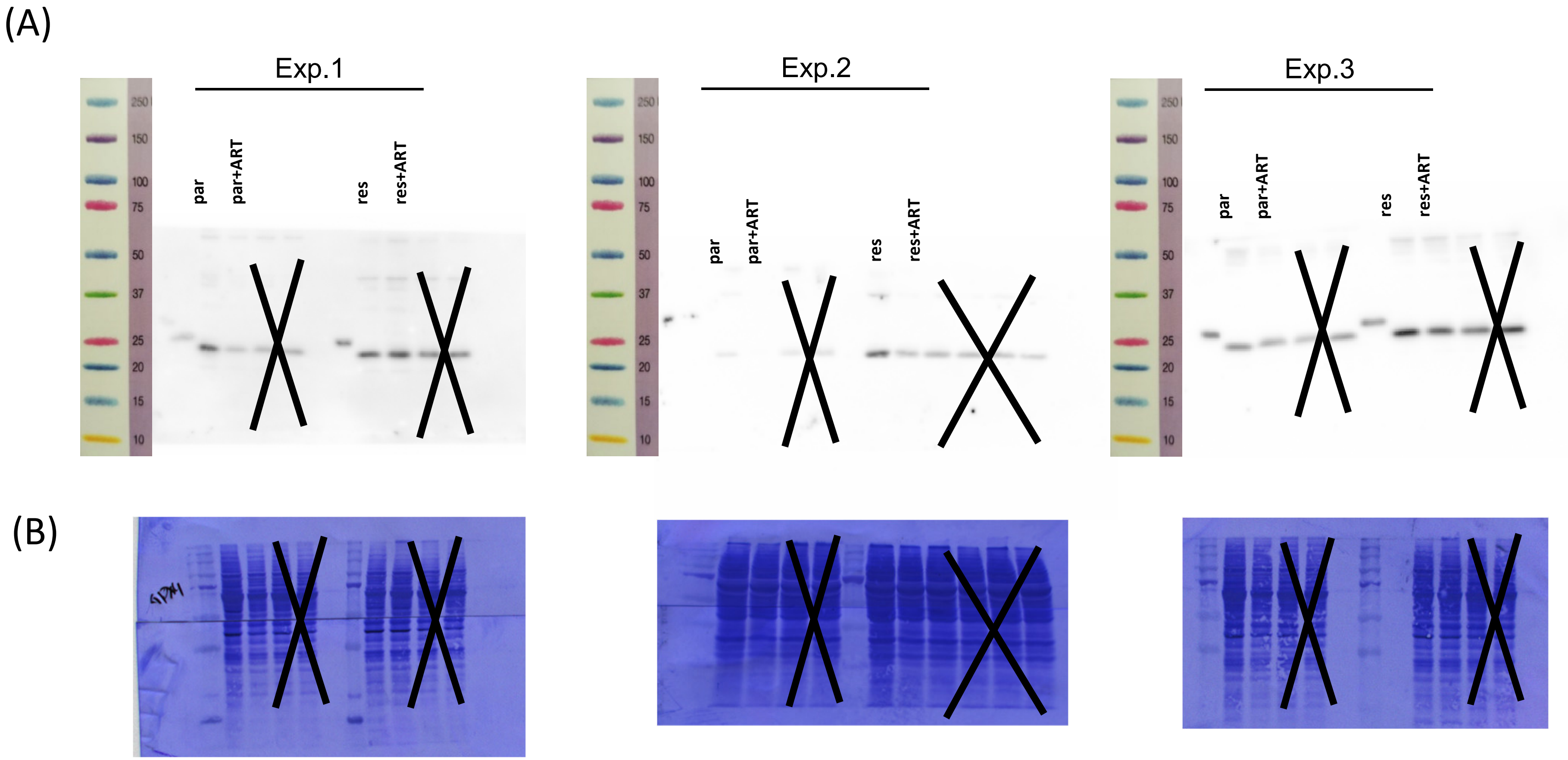

Figure S3: Detailed information about Figure 8 - Protein expression profile of ferroptosis associated protein in parental and resistant DU145 cells. Protein expression of GPX4 (A), corresponding Coomassie blue staining of total protein (B).
